# Supplementary material for: CMT3 and SUVH4/KYP silence the exonic Evelknievel retroelement to allow for reconstitution of CMT1 mRNA
Source: Epigenetics Chromatin. 2018 Nov 16;11:69. doi: 10.1186/s13072-018-0240-y (PMC6238269; doi:10.1186/s13072-018-0240-y)
Supplement: Supplementary file 1 — Additional file 1. Figure S1: The distribution of sRNAs along CMT1-Evelknievel locus in Ler inflorescence tissues. Figure S2: Chop PCR demonstrating erasure of CHG methylation in EK coding region (EKcr) in cmt3 and kyp2 mutants. Figure S3: Intron 12 retention. Figure S4: Analysis of chimeric CMT1-EK RNA. Figure S5: Confirmation of the full-length CMT1 RNA by sequencing. Table S1: List of primers used in the present work. Bisulfite sequencing (BS-seq) data. [file 13072_2018_240_MOESM1_ESM.docx]

**Additional file 1**

**CMT3 and SUVH4/KYP silence the exonic retroelement Evelknievel to allow for reconstitution of *CMT1* mRNA**

Narendra Singh Yadav^1§^, Janardan Khadka^1§^, Katherine Domb^2^, Assaf Zemach^2^ and Gideon Grafi^1^*

^1^French Associates Institute for Agriculture and Biotechnology of Drylands, Jacob BlausteinInstitutes for Desert Research, Ben-Gurion University of the Negev, Midreshet Ben Gurion 84990, Israel. ^2^The School of Plant Sciences and Food Security, Tel-Aviv University, 69978, Tel Aviv, Israel


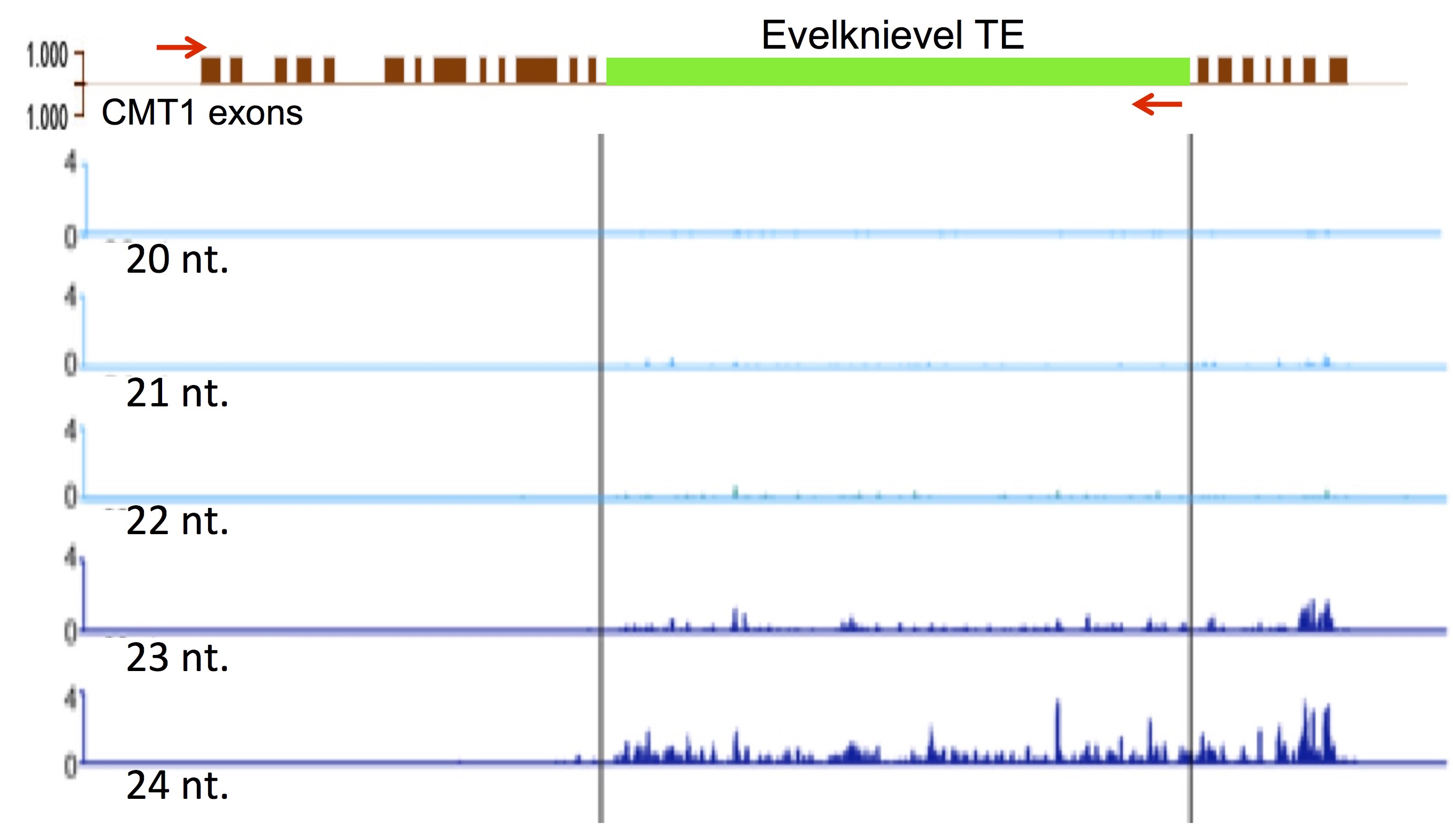


Figure S1. The distribution of sRNAs in Ler inflorescence tissues along the Evelknievel (EK)-3’ CMT1 sequence. Note that sRNA of 24 nt are most prominent and their distribution perfectly match the pattern of DNA methylation shown in Fig. 1a (main text). A schematic representation of the CMT1-EK locus is shown at the top. Arrows indicate the transcriptional direction of *CMT1* and EK.

**sRNA analysis**

Publicly available sRNA data of Ler inflorescence tissues were downloaded from GEO ([GSE60826](https://www.ncbi.nlm.nih.gov/geo/query/acc.cgi?acc=GSE60826), Wang et al., 2015). Adaptors were trimmed using TrimGalore software, and 18-30 nt. trimmed reads were aligned to the reference genome using Bowtie-2 with no mismatches and reporting only uniquely mapped reads (-v 0 -m 1). The numbers of 20-24 nt. reads were calculated in 1-bp genomic windows and normalized to library size (RPM). For the examined inflorescent tissues sRNA reads of two samples were combined.

Wang X, Zhang S, Dou Y, Zhang C, Chen X, Yu B, Ren G. Synergistic and independent actions of multiple terminal nucleotidyl transferases in the 3' tailing of small RNAs in Arabidopsis. PLoS Genet. 2015;11:e1005091.


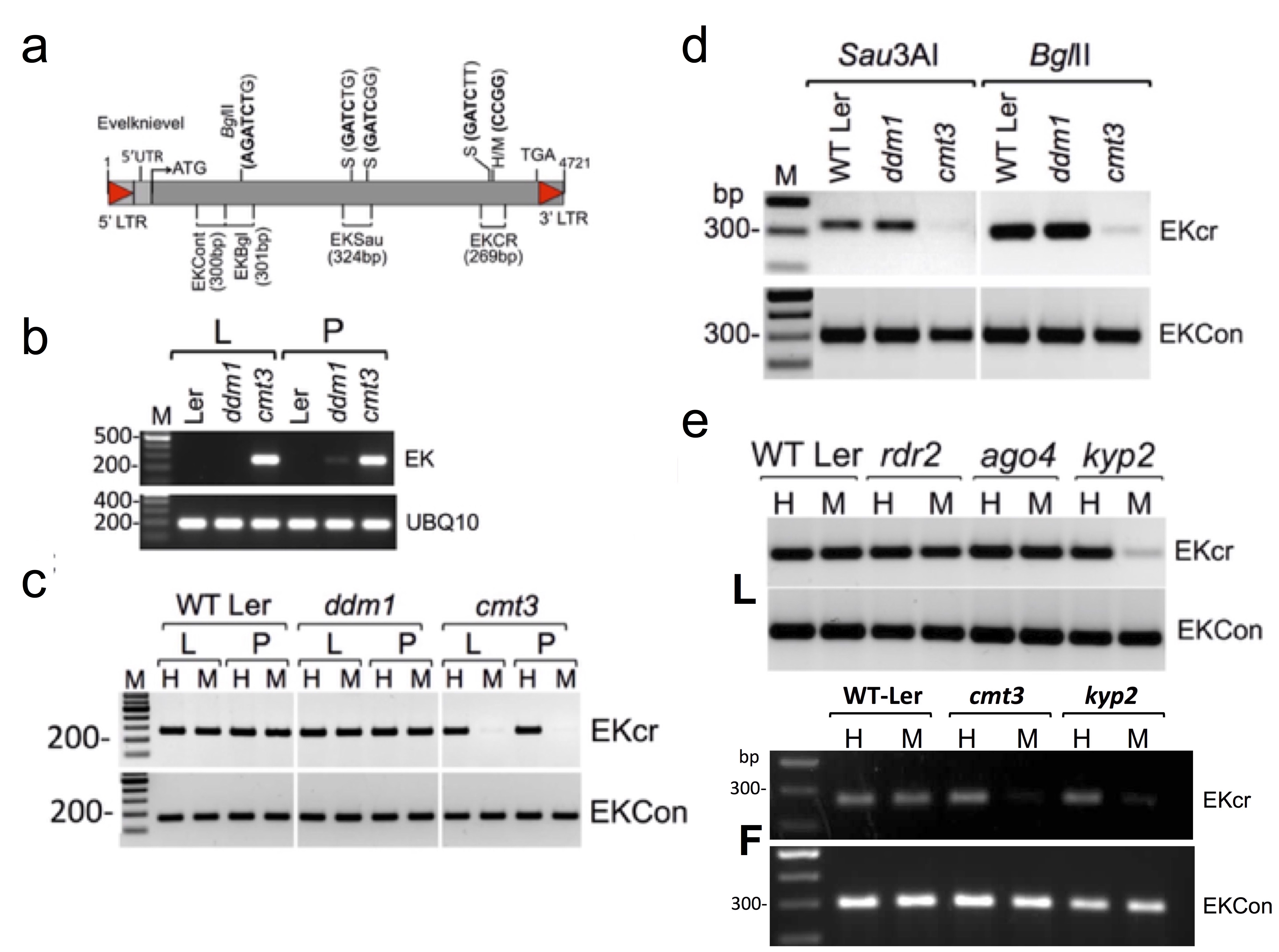


Figure S2. Reduction in EK coding region (EKcr) CHG methylation in cmt3 and kyp2 mutants. (**a**) Schematic representation of the EK retroelement. LTRs are marked by red boxes. The EK coding regions used for monitoring DNA methylation by the indicated methylation sensitive restriction enzymes are shown with their expected sizes of PCR products. Control (EKCont) is an EK region lacking sites for the indicated restriction enzymes used as a reference in chop PCR experiments. S, *Sau*3AI; HM, *Hpa*II and *Msp*I. (**b**) EK is not activated following exposure to stress (protoplasting). cDNAs were prepared from RNA extracted from Leaves (L) and protoplasts (P) of WT-Ler, *ddm1* and *cmt3* mutant and subjected to PCR to amplify EK coding region (EKcr). UBQ10 was used as a reference. M indicates DNA molecular size markers. (**c**) Reduction in EKcr CHG methylation in *cmt3* mutants. Genomic DNAs prepared from leaves (L) and protoplasts (P) of the indicated lines were digested with methylation sensitive restriction enzymes *Hpa*II (H) and *Msp*I (M) and subjected to PCR to amplify EKcr. EKCon is a control of the EK region with no CCGG sites. (**d**) Chop PCR using methylation sensitive enzymes *Bgl*II and *Sau*3AI confirming reduction in EKcr CHG methylation in *cmt3* mutant. Genomic DNAs prepared from leaves of the indicated lines were digested with methylation sensitive restriction enzymes *Sau*3AI (left panel) or *Bgl*II (right panel) and subjected to PCR to amplify EKcr. EKCont is a control of the EK region with no restriction sites for the indicated enzymes. (**e**) Chop-PCR showing reduction in EKcr CHG methylation in *kyp2* and *cmt3* mutants. Genomic DNAs prepared from leaves (L, upper panel) or flowers (F, lower panel) of the indicated lines were digested with methylation sensitive restriction enzymes *Hpa*II (H) and *Msp*I (M) and subjected to PCR to amplify EKcr. EKCont is a control of the EK region with no CCGG sites.

**
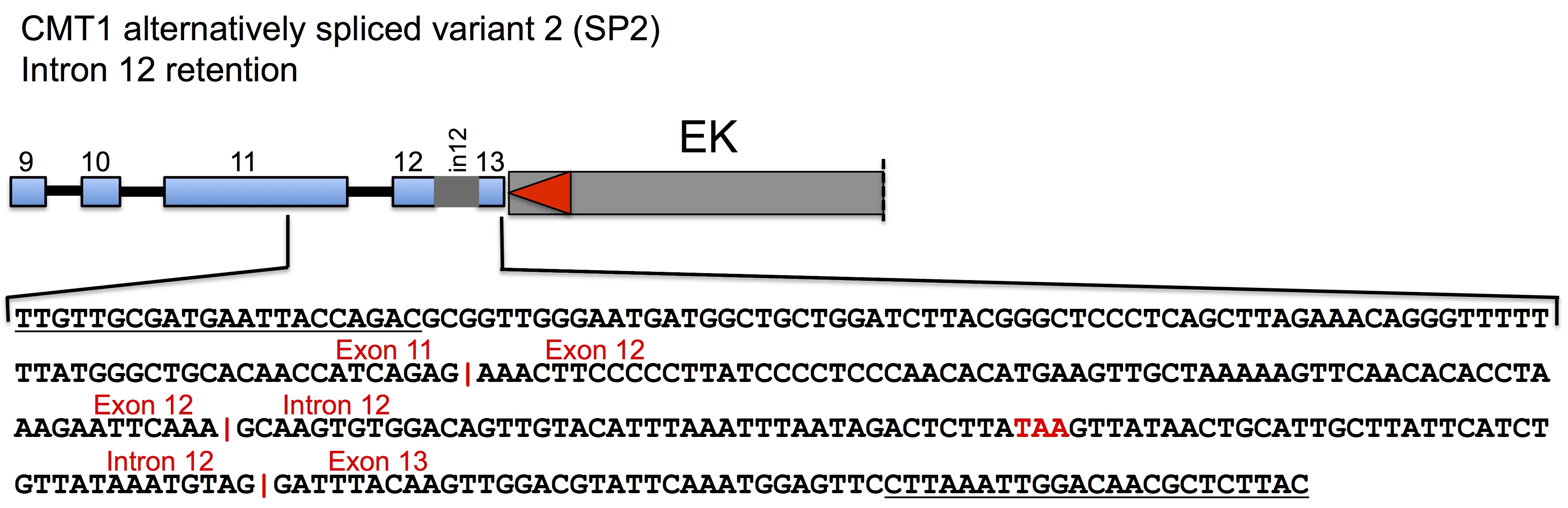
**

Figure S3. Intron 12 retention. Schematic representation of the alternatively spliced region of CMT1 downstream from EK insertion site. Red arrowhead indicates the EK 3’LTR. *CMT1* exons are numbered and shown as blue boxes and introns as black lines. The DNA sequence of SP2 is shown with primers used underlined. The exons and the retained intron 12 (in12) are indicated. The borders between exon-exon and exon-intron are marked by vertical red bar. The stop codon TAA within intron 12 is shown in red.


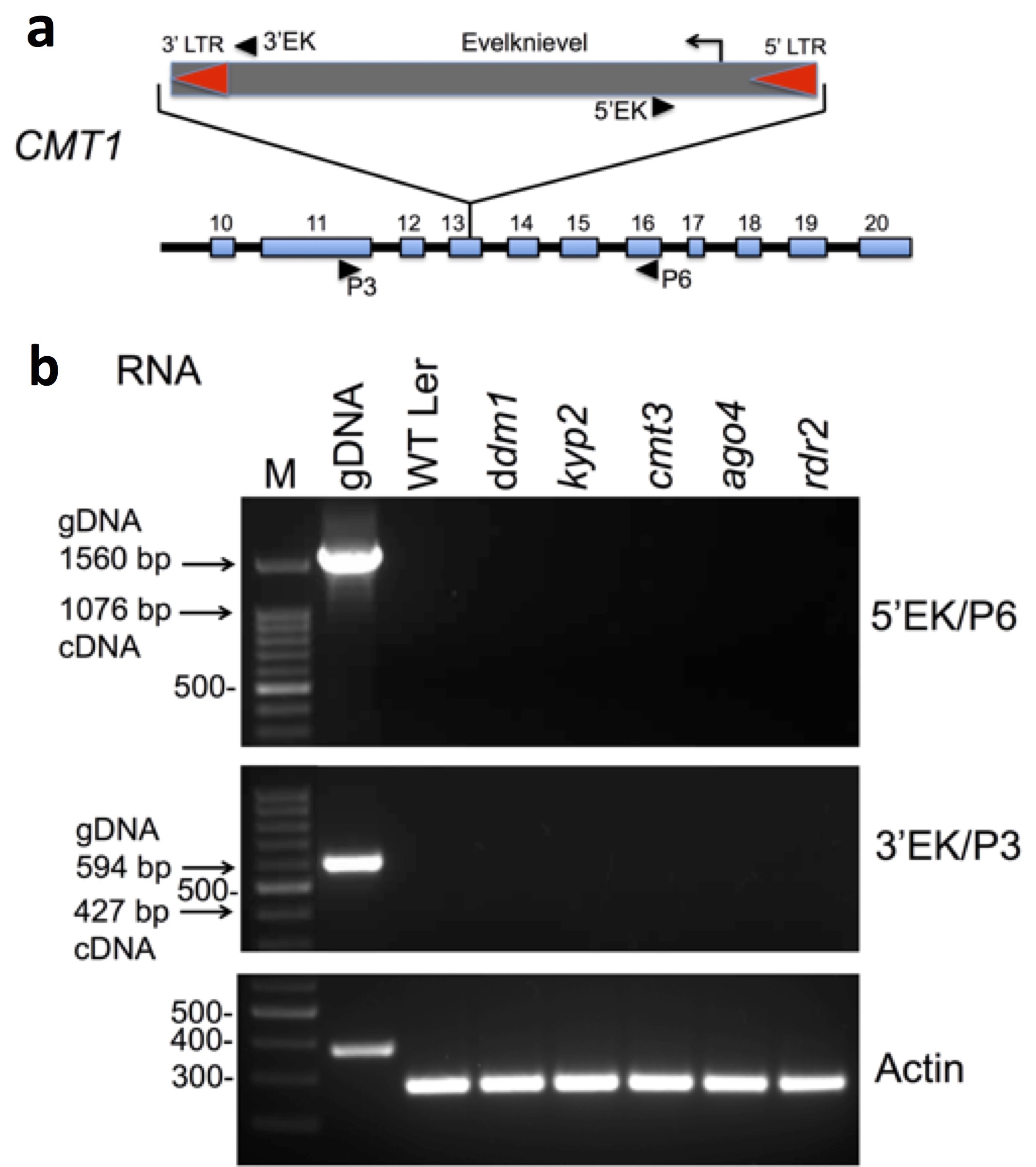


Figure S4. Analysis of chimeric CMT1-EK RNA. (**a**) Schematic representation of the EK insertion site within *CMT1* gene*.* The position of primers P6-5’EK and P7-3’EK used to check chimeric RNA are indicated by arrowheads. (**b**) Lack of chimeric CMT1-EK RNAs. PCR products derived from WT Ler and from the indicated mutant cDNAs were separated on agarose gel stained with ethidium bromide. The expected sizes and positions of PCR products derived from genomic DNA (gDNA) and from cDNAs are indicated by arrows. Actin was used as a reference. M indicates the DNA size markers.


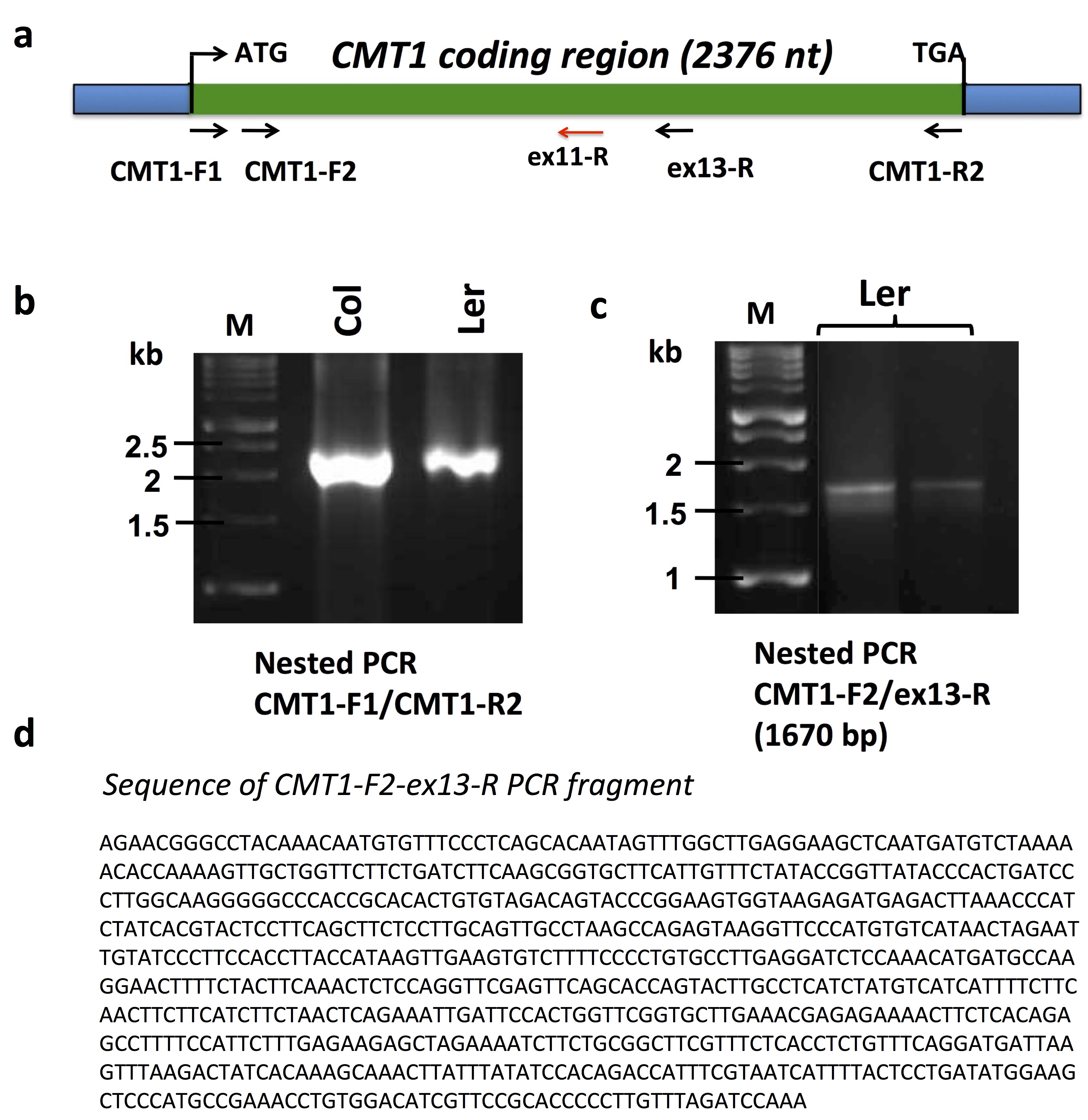


Figure S5. Confirmation of the full-length CMT1 RNA. Schematic representation of the *CMT1* RNA. (**a**) Schematic representation of the CMT1 RNA. The positions of primers used are indicated (primers sequence is given in Table S1). (**b**) Re-amplification of the nested PCR product derived from Col and Ler ecotypes. (**c**) Nested PCR of the Ler PCR product using CMT1-F2 and ex13R primers. The PCR fragment was separated on 1% agarose gel, eluted from the gel and sequenced. (**d**) The nucleotide sequence of the CMT1-F2-ex13-R fragment was subjected to sequencing using internal primer ex11-R. The sequence matches the *CMT1* RNA.

**Table S1. Primers used in this study**

| **Oligo Name** | **Sequence (5' to 3')** | **Used for** |
| --- | --- | --- |
| EK-RTF | TTCGCAACTGCTCGATATTCT | EK expression analysis and CHOP PCR for *Hpa*II/*Msp*I |
| EK-RTR | CACTGTCCAACCGATCTTCAT |  |
| EKSau3-F | CACAAGAGATTCTTCACTCAACG | CHOP PCR for *Sau*3AI |
| EKSau3-R | CTATGTCTCGATAGAGCTACTG |  |
| EKBgl-F | AGGTTAGAAGTGAGGTGATGCGTC | CHOP PCR for *Bgl*II |
| EKBgl-R | GTAACGGTCGCAACAACTCTC |  |
| EKcont-F | ACTATTCTGCCATGAAGTGTTAC | Control for CHOP PCR |
| EKcont-R | CTTGCGCTACTTGGAAAGGCTC |  |
| bsEK5LTR-F1 | TTAGTAAGGTGGAAGTGGAG | Bisulphite sequencing of 5'LTR |
| bsEx14-R1 | AACTAATAAAATTCTCAAACTCT |  |
| bsEK5LTR-F2 | GGAGATAGATATTGGGGAGGTTGAA |  |
| bsEx14-R2 | ATAATCCATCACATCATTTACCACATA |  |
| bsEKcr-F1 | GTGGGGAATGTTGTGGATTTGG | Bisulphite sequencing of EK coding region |
| bsEKcr-R1 | CCTTCTATCACACCTCCATCA |  |
| bsEKcr-F2 | GGTTGTGGGTTTGAGGAATGTG |  |
| bsEKcr-R2 | CCATCAAATCCTCCTCTCTCAC |  |
| UBQ10-F | TTTGTTAAGACTCTCACCGGAAAGACA | Control of RT-PCR |
| UBQ10-R | GAGGGTGGATTCCTTCTGGATATTGTA |  |
| ACT-F | TGGCCGATGGTGAGGATATT | Control of RT-PCR |
| ACT-R | GCTCGTTGTAGAAAGTGTGATG |  |
| 18A-F | TGGTTTGCAGCCTAATAAACCT | AtCOPIA18A expression analysis |
| 18A-R | GAGACTCACGGGTACGTCTTTC |  |
| Solo-F | AACTAACGTCATTACATACACATCTTG | Solo LTR expression analysis  (Moissiard et al 2012) |
| Solo-R | AATTAGGATCTTGTTTGCCAGCTA |  |
| ex10-F (P1) | GCAAGGAGAAGCTGAAGGAG | Upstream *CMT1* expression analysis |
| ex11-R (P2) | ATGACGTGCCAGGAAACC |  |
| ex11-F (P3) | TTGTTGCGATGAATTACCAGAC | Upstream *CMT1* expression analysis |
| ex13-R (P4) | GTAAGAGCGTTGTCCAATTTAAG |  |
| ex14-F (P5) | GTGGCAAATGATGTGATGGAT | Downstream *CMT1* expression analysis |
| ex16-R (P6) | ACTAAAACACCAGGCATGTCG |  |
| ex13-F (P7) | AAATTGGACAACGCTCTTAC | For entire EK splice out experiment  Using ex16-R(P6) as a reverse primer |
| AtMu1-F | GCAAGAGCTGTGGTGAAG | AtMu1 expression analysis |
| AtMu1-R | TGCTTGAGAAGGTTGTGTGAT |  |
| CMT1-F1 | ATGGCAGCGAGAAACAAACAAAA | CMT1 full-length and validation |
| CMT1-F2 | CTGAGCCAGAGTCAGATCTGTGCTTC |  |
| CMT1-R2 | TCAGATTTGATCCTTTGCCTGC |  |

**Bisulfite Sequencing Data**

Bisulfite sequencing of Evelknievel coding region and the 5’LTR-CMT1 region in WT Ler and various epigenetic mutants.

**Part I: EK5’LTR-CMT1 region**

**Sequence used for bisulfite sequencing** (Primers-underlined, 5’LTR-red colored, start codon at reverse strand - yellow highlighted, CMT1-purple colored)

TCAGCAAGGTGGAAGTGGAGACGGCGATGGTCTCGGAGACAGATACTGGGGAGGTTGAATCAGACATCGTGAAAGAAGAGAGAGAAAAAGAGAGATGGAAAAGAGAAGAAGAAAGAGCGGCGGTAGGGAATTTTTTTTTCTTTTTTTTTTCTTTAGATTTAGGTATCGACAAGCTCTGATACCATGTATGAAATGAGGCTCAATAGTTTATTACATAGATGTTTGTACAATATATATAGTGATTACAAGAGTAATTAGGTATCTATAATTTACAATTATATCCTTAGTCATTTCTCCCTTACAGGCTGATGCTATTAGTGATCTGCCACCGGTGTGTATCTAAACTTCAGAACTGCCATTGATGATTATGTTACAATATATCTGTCTGATCACTGCTCTCTTAGTGCTGCAGGTGACAAATTATGTGGCAAATGATGTGATGGATTATAATGATGCTGCTCCTAAGACAGAGTTTGAGAACTTTATTAGCC

**WTLer- EK5’LTR-CMT1**

LEP13

AATCAGATATCGTGAAAGAAGAGAGAGAAAAAGAGAGATGGAAAAGAGAAGAAGAAAGAGCGGCGGTAGGGAATTTTTTTTTTTTTTTTTTTTCTTTAGATTTAGGTATCGACAAGTTCTGATATTATGTATGAAATGAGGTTCAATAGTTTATTATATAGATGTTTGTACAATATATATAGTGATTACAAGAGTAATTAGGTATCTATAATTTACAATTATATTCTTAGTTATTTCTTCCTTACAGGCTGATGCTATTAGTGATCTGTTACCGGTGTGTATCTAAATTTCAGAATTGTTATTGATGATTATGTTACAATATATCTGTCTGATTACTGCTCTCTTAATGCTGCAGGTGACAAAT

LEP14

AATTAGATATCATGAAAGAAGAGAGAGAAAAAGAGAGATGGAAAAGAGAAGAAGAAAGAGCGGCGGTAGGGAATTTTTTTTTTTTTTTTTTTTTTTTAGATTTAGGTATCGACAAGTTCTGATATTATGTATGAAATGAGGTTCAATAGTTTATTATATAGATGTTTGTATAATATATATAGTGATTATAAGAGTAATTAGGTATTTATAATTTACAATTATATTTTTAGTTATTTCTCTCTTACAGGTTGATGCTATTAGTGATCTGTTACCGGTGTGTATCTAAATTTCAGAACTGTTATTGATGATTATGTTATAATATATCTGTCTGATTACTGTTCTTTTAATGCTGCAGGTGATAAAT

LEP15

AATCAGATATCGTGAAAGAAGAGAGAGAAAAAGAGAGATGGAAAAGAGAAGAAGAAAGAGCGGCGGTAGGGAATTTTTTTTTCTTTTTTTTTTTTTTAGATTTAGGTATCGACAAGTTCTGATATTATGTATGAAATGAGGCTCAATAGTTTATTATATAGATGTTTGTATAATATATATAGTGATTACAAGAGTAATTAGGTATCTATAATTTACAATTATATTCTTAGTTATTTTTTTCTTATAGGCTGATGCTATTAGTGATCTGTTATCGGTGTGTATTTAAATTTCAGAACTGTTATTGATGATTATGTTACAATATATCTGTCTGATTACTGTTCTCTTAATGCTGCAGGTGATAAAT

LEP16

AATTAGACATCGTGAAAGAAGAGAGAGAAAAAGAGAGATGGAAAAGAGAAGAAGAAAGAGCGGCGGTAGGGAATTTTTTTTTTTTTTTTTTTTTTTCTTTAGATTTAGGTATCGACAAGCTCTGATATTATGTATGAAATGAGGTTCAATAGTTTATTATATAGATGTTTGTACAATATATATAGTGATTACAAGAGTAATTAGGTATTTATAATTTACAATTATATTCTTAGTCATTTTTCCCTTACAGGCTGATGCTATTAGTGATCTGTTACCGGTGTGTATTTAAATTTCAGAACTGTTATTGATGATTATGTTATAATATATCTGTCTGATTATTGTTTTTTTAATGTTGTAGGTGATAAAT

LEP18

AATTAGACATCGTGAAAGAAGAGAGAGAAAAAGAGAGATGGAAAAGAGAAGAAGAAAGAGCGGCGGTAGGGAATTTTTTTTTTTTTTTTTTTTCTTTAGATTTAGGTATCGACAAGCTTTGATATTATGTATGAAATGAGGCTCAATAGTTTATTATATAGATGTTTGTACAATATATATAGTGATTACAAGAGTAATTAGGTATCTATAATTTACAATTATATTTTTAGTTATTTTTTTCTTACAGGCTGATGCTATTAGTAATCTGCCACCGGTGTGTATCTAAATTTCAGAACTGTCATTGATGATTATGTTACAATATACCTGTCTGATTACTGTTCTCTTAATGTTGCAGGTGATAAAT

LEP19

AATCAGACATCGTGAAAGAAGAGAGAGAAAAAGAGAGATGGAAAAGAGAAGAAGAAAGAGCGGCGGTAGGGAATTTTTTTTTTTTTTTTTTTTTTTTTAGATTTAGGTATCGACAAGTTCTGATATTATGTATGAAATGAGGTTCAATAGTTTATTATATAGATGTTTGTACAATATATATAGTGATTACAAGAGTAATTAGGTATCTATAATTTACAATTATATTTTTAGTCATTTCTTTCTTACAGGCTGATGCTATTAGTGATCTGTCACCGGTGTGTATCTAAATTTCAGAACTGTTATTGATGATTATGTTACAATATATTTGTCTGATTACTGCTCTCTTAATGCTGCAGGTGACAAAT

LEP21

AATCAGATATCGTGAAAGAAGGGAGAGAAAAAGAGAGATGGAAAAGAGAAGGAGAAAGAGCGGCGGTAGGGAATTTTTTTTTTTTTTTTTTTTTTTTTAGATTTAGGTATCGACAAGTTCTGATATTATGTATGAAATGAGGCTCAATAGTTTATTATATAGATGTTTGTATAATATATATAGTGATTACAAGAGTAATTAGGTATCTATAATTTACAATTATATTCTTAGTTATTTTTTTTTTACAGGCTGATGCTATTAGTGATCTGTCATCGGTGTGTATTTAAATTTTAGAACTGTTATTGATGATTATGTTATAATATATCTGTCTGATTACTGTTTTTTTAATGTTGCAGGTGATAAAT

LEP22

AATCAGATATCGTGAAAGAAGAGAGAGAAAAAGAGAGATGGAAAAGAGAAGAAGAAAGAGCGGCGGTAGGGAATTTTTTTTTTTTTTTTTTTTCTTTAAATTTAGGTATCGACAAGTTCTGATATTATGTATGAAATGAGGTTCAATAGTTTATTACATAGATGTTTGTACAATATATATAGTGATTACAAGAGTAATTAGGTATCTATAATTTACAATTATATTCTTAGTTATTTCTTCCTTACAGGCTGATGCTATTAGTGATCTGTTACCGGTGTGTATCTAAATTTCAAAATTGTTATTGATGATTATGTTACAATATATCTGTCTGATTACTGCTCTCTTAATGCTGCAGGTGACAAAT

EPL57

AATCAGATATCGTGAAAGAAGAGAGAGAAAAAGAGAGATGGAAAAGAGAAGAAGAAAGAGCGGCGGTAGGGAATTTTTTTTTTTTTTTTTTTTTTAAAATTAAGGATCCACCAATTCCGAAATTATTTATGAAAAGAAGTTTAATAATTTATTATATAAATTTTTGTATAAAATATATTATGATTATAAAAATAATTAAGTATCCATAAATTACCAATTTATTCCTAATTTTTTCCTTCCTACCAGGCGAAGCCATTAATGATTCGGTATCCGTGTGTATCCAAATTTCCAAACTTGTATTTAATAATATTTTTTAATATACCCGGCCGAATACCGGTTTTTTTATGGTTCCAGGGAAAAAT

EPL58

AATCAGATATCGTGAAAGAAGAGAGAGAAAAAGAGAGATGGAAAAGAGAAGAAGAAAGAGCGGCGGTAGGGAATTTTTTTTTTTTTTTTTTTTCTTTAGATTTAGGTATCGACAAGTTCTGATATTATGTATGAAATGAGGTTCAATAGTTTATTATATAGATGTTTGTATAATATATATAGTGATTATAAGAGTAATTAGGTATTTATAATTTATAATTATATTTTTAGTTATTTTTTTCTTACAGGCTGATGCTATTAGTGATCTGTTATCGGTGTGTATTTAAATTTTAGAATTGTTATTGATGATTATGTTATAATATATTTGTTTGATTACTGTTCTCTTAATGTTGCAGGTGACAAAT

EPL60

AATTAGATATTGTGAAAGAAGAGAGAGAAAAAGAGAGATGGAAAAGAGAAGAAGAAAGAGCGGCGGTAGGGAATTTTTTTTTTTTTTTTTTTTTTTAGATTTAGGTATCGACAAGTTCTGATATTATGTATGAAATGAGGTTTAATAGTTTATTATATAGATGTTTGTATAATATATATAGTGATTATAAGAGTAATTAGGTATTTATAATTTATAATTATATTTTTAGTTATTTTTTTCTTACAGGCTGATGTTATTAGTGATCTGTTACCAGTGTGTATTTAAATTTTAGAACTGTTATTGATGATTATGTTATAATATATCTGTCTGATTACTGTTCTCTTAATGCTGCAGGTGATAAAT

EPL61

AATCAGATATCGTGAAAGAAGAGAGAGAAAAAGAGAGATGGAAAAGAGAAGAAGAAAGAGCGGCGGTAGGGAATTTTTTTTTTTTTTTTTTTTTTTTTTAGATTTAGGTATCGACAAGTTCTGATATTATGTATGAAATGAGGTTTAATAGTTTATTATATAGATGTTTGTATAATATATATAGTGATTATAAAAGTAATTAGGTATTTATAATTTATAATTATATTTTTAGTTATTTTTTTCTTACAGGTTGATGCTATTAGTGATCTGTTACCGGTGTGTATCTAAATTTCAGAACTGTTATTGATGATTATGTTATAATATATTTGTTTGATTATTGTTTTTTTAATGCTGCAGGTGATAAAT

EPL63

AATCAGATATCGTGAAAGAAGAGAGAGAAAAAGAGAGATGGAAAAGAGAAGAAGAAAGAGCGGCGGTAGGGAATTTTTTTTTTTTTTTTTTTTTTTTTAGATTTAGGTATCGACAAGTTCTGATATTATGTATGAAATGAGGTTCAATAGTTTATTATATAGATGTTTGTATAATATATATAGTGATTACAAGAGTAATTAGGTATTTATAATTTACAATTATATTTTTAGTTATTTTTTCCTTACAGGCTGATGCTATTAGTGATCTGCCACCGGTGTGTATCTAAATTTCAGAACTGTTATTGATGATTATGTTACAATATATCTGTCTGATTACTGTTCTCTTAATGCTGCAGGTGACAAAT

EPL64

AATTAGATATCGTGAAAGAAGAGAGAGAAAAAGAGAGATGGAAAAGAGAAGAAGAAAGAGCGGCGGTAGGGAATTTTTTTTTTTTTTTTTTTTTTTTAGATTTAGGTATCGACAAGTTCTGATATTATGTATGAAATGAGGTTCAATAGTTTATTATATAGATGTTTGTACAATATATATAGTGATTACAAGAGTAATTAGGTATCTATAATTTACAATTATATTTTTAGTTATTTTTTTCTTATAGGTTGATGCTATTAGTGATCTGTTACCGGTGTGTATCTAAATTTCAGAATTGTTATTGATGATTATGTTATAATATATCTGTCTGATTATTGTTTTCTTAATGCTGCAGGTGATAAAT

EPL65

AATCAGACATCGTGAAAGAAGAGAGAGAAAAAGAGAGATGGAAAAGAGAAGAAGAAAGAGCGGCGGTAGGGAATTTTTCTTTTTTTTTTTTTTTTTAGATTTAGGTATCGACAAGTTCTGATATTATGTATGAAATGAGGTTCAATAGTTTATTATATAGATGTTTGTACAATATATATAGTGATTACAAGAGTAATTAGGTATCTATAATTTACAATTATATTTTTAGTTATTTTTTTCTTACAGGCTGATGCTATTAGTGATCTGTTACCGGTGTGTATCTAAATTTCAGAACTGTCATTGATGATTATGTTACAATATATCTGTCTGATTACTGCTCTCTTAATGCTGCAGGTGACAAAT

EPL67

AATCAGATATCGTGAAAGAAGAGAGAGAAAAAGAGAGATGGAAAAGAGAAGAAGAAAGAGCGGCGGTAGGGAATTTTTTTTTTTTTTTTTTTTTTTAGATTTAGGTATCGACAAGTTCTGATATTATGTATGAAATGAGGTTCAATAGTTTATTATATAGATGTTTGTACAATATATATAGTGATTACAAGAGTAATTAGGTATCTATAATTTACAATTATATTTTTAGTTATTTTTTTCTTACAGGCTGATGCTATTAGTGATCTGTTACCGGTGTGTATCTAAATTTCAGAACTGTTATTGATGATTATGTTACAATATATCTGTCTGATTACTGTTCTCTTAATGCTGCAGGTGATAAAT

EPL68

AATCAGACATCGTGAAAGAAGAGAGAGAAAAAGAGAGATGGAAAAGAGAAGAAGAAAGAGCGGCGGTAGGGAATTTTTTTTTTTTTTTTTTTTTTTTTAGATTTAGGTATCGACAAGTTCTGATATTATGTATGAAATGAGGTTCAATAGTTTATTATATAGATGTTTGTACAATATATATAGTGATTACAAGAGTAATTAGGTATCTATAATTTACAATTATATTTTTAGTTATTTTTTTCTTACGGGCTGATGCTATTAGTGATCTGTTACCGGTGTGTATCTAAATTTCAGAACTGTCATTGATGATTATGTTACAATATATCTGTCTGATTACTGCTCTCTTAATGCTGCAGGTGACAAAT

EPL70

AATCAGACATCGTGAAAGAAGAGAGAGAAAAAGAGAGATGGAAAAGAGAAGAAGAAAGAGCGGCGGTAGGGAATTTTTTTTTTTTTTTTTTTTTTTTTTAGATTTAGGTATCGACAAGTTCTGATATTATGTATGAAATGAGGCTCAGTAGTTTATTATATAGATGTTTGTACAATATATATAGTGATTACAAGAGTAATTAGGTATCTATAATTTACAATTATATTTTTAGTTATTTCTTTCTTATAGGCTGATGCTATTAGTGATCTGTCACCGGTGTGTATCTAAATTTTAGAATTGTTATTGATGATTATGTTACAATATATCTGTCTGATCATTGTTTTCTTAATGTTGCAGGTGACAAAT

***ddm1*- EK5’LTR-CMT1**

EPD71

AATCAGACATCGTGAAAGAAGAGAGAGAAAAAGAGAGATGGAAAAGAGAAGAAGAAAGAGCGGCGGTAGGGAATTTTTTTTTTTTTTTTTTTTTTTTTAGATTTAGGTATCGACAAGTTCTGATATTATGTATGAAATGAGGTTTAATAGTTTATTATATAGATGTTTGTATAATATATATAGTGATTACAAGAGTAATTAGGTATTTATAATTTATAATTATATTTTTAGTTATTTTTTTTTTACAGGTTGATGCTATTAGTGATCTGTTACCGGTGTGTATTTAAATTTCAGAACTGTTATTGATGATTATGTTACAATATATCTGTTTGATTACTGTTTTTTTAATGTTGTAGGTGATAAAT

EPD72

AATTAGACATTGTGAAAGAAGAGAGAGAAAAAGAGAGATGGAAAAGAGAAGAAGAAAGAGCGGCGGTAGGGAATTTTTTTTTTTTTTTTTTTTTTTTTAGATTTAGGTATCGACAAGTTCTGATATTATGTATGAAATGAGGTTTAATAGTTTATTATATAGATGTTTGTATAATATATATAGTGATTACAAGAGTAATTAGGTATTTATAATTTATAATTATATTTTTAGTTATTTTTTTCTTACAGGCTGATGCTATTAGTGATCTGCCACCGGTGTGTATCTAAATTTCAGAATTGTTATTGATGATTATGTTACAATATATCTGTCTGATTATTGTTCTCTTAATGCTGCAGGTGATAAAT

EPD73

AATCAGATATCGTGAAAGAAGAGAGAGAAAAAGAGAGATGGAAAAGAGAAGAAGAAAGAGCGGCGGTAGGGAATTTTTTTTTTTTTTTTTTTTTTAGATTTAGGTATCGACAAGTTCTGATATTATGTATGAAATGAGGTTCAATAGTTTATTATATAGATGTTTGTACAATATATATAGTGATTACAAGAGTAATTAGGTATTTATAATTTATAATTATATTTTTAGTTATTTTTTTTTTTACAGGCTGATGCTATTAGTGATCTGTTACCGGTGTGTATTTAAATTTCAGAACTGTTATTGATGATTATGTTACAATATATCTGTTTGATTACTGTTTTTTTAATGTTGTAGGTGATAAAT

EPD74

AATCAGATATCGTGAAAGAAGAGAGAGAAAAAGAGAGATGGAAAAGAGAAGAAGAAAGAGCGGCGGTAGGGAATTTTTTTTTTTTTTTTTTTTTTTTTTAGATTTAGGTATCGACAAGTTCTGATATTATGTATGAAATGAGGTTCAATAGTTTATTATATAGATGTTTGTACAATATATATAGTGATTACAAGAGTAATTAGGTATCTATAATTTATAATTATATTTTTAGTTATTTTTTTTTTACAGGTTGATGTTACTAGTGATTTGTTACCGGTGTGTATCTAAATTTCAGAACTGTTATTGATGATTATGTTACAATATATCTGTCTGATTACTGTTCTCTTAATGTTGCAGGTGACAAT

EPD75

AATCAGATATTGTGAAAGAAGAGAGAGAAAAAGAGAGATGGAAAAGAGAAGAAGAAAGAGCGGCGGTAGGGAATTTTTTTTTTTTTTTTTTTTTTTTTAGATTTAGGTATCGACAAGTTCTGATATTATGTATGAAATGAGGTTTAATAGTTTATTATATAGATGTTTGTATAATATATATAGTGATTACAAGAGTAATTAGGTATCTATAATTTATAATTATATTTTTAGTTATTTTTTTCTTATAGGTTGATGCTATTAGTGATTTGTTATTGGTGTGTATTTAAATTTTAGAATTGTTATTGATGATTATGTTATAATATATCTGTCTGATTACTGTTTTTTTAATGCTGCAGGTGATAAAT

EPD76

AATTAGATATCGTGAAAGAAGAGAGAGAAAAAGAGAGATGGAAAAGAGAAGAAGAAAGAGTGGTGGTAGGGAATTTTTTTTTTTTTTTTTTTTTTAGATTTAGGTATCGATAAGTTCTGATATTATGTATGAAATGAGGTTTAATAGTTTATTATATAGATGTTTGTATAATATATATAGTGATTACAAGAGTAATTAGGTATTTATAATTTATAATTATATTTTTAGTTATTTTTTTCTTACAGGCTGATGCTATTAGTGATCTGTTACCGGTGTGTATTTAAATTTTAGAACTGTTATTGATGATTATGTTACAATATATCTGTCTGATTATTGTTTTTTTAATGTTGTAGGTGATAAAT

EPD77

AATCAGATATCGTGAAAGAAGAGAGAGAAAAAGAGAGATGGAAAAGAGAAGAAGAAAGAGCGGCGGTAGGGAATTTTTTTTTTTTTTTTTTTTCTTTAGATTTAGGTATCGATAAGTTCTGATATTATGTATGAAATGAGGTTCAATAGTTTATTATATAGATGTTTGTACAATATATATAGTGATTACAAGAGTAATTAGGTATTTATAATTTATAATTATATTTTTAGTTATTTTTTCCTTACAGGCTGATGCTATTAGTGATTTGTTACCGGTGTGTATCTAAATTTCAGAATTGTTATTGATGATTATGTTACAATATATCTGTCTGATTACTGTTTTCTTAATGCTGTAGGTGACAAAT

EPD80

AATCAGATATCGTGAAAGAAGAGAGAGAAAAAGAGAGATGGAAAAGAGAAGAAGAAAGAGCGGCGGTAGGGAATTTTTTTTTTTTTTTTTTTTTTTTAGATTTAGGTATCGACAAGTTCTGATATTATGTATGAAATGAGGTTTAATAGTTTATTATATAGATGTTTGTATAATATATATAGTGATTATAAGAGTAATTAGGTATTTATAATTTATAATTATATTTTTAGTTATTTTTTTTTTACAGGCTGATGTTATTAGTGATCTGTTACCGGTGTGTATCTAAATTTCAGAACTGTTATTGATGATTATGTTATAATATATCTGTCTGATTACTGTTCTCTTAATGTTGCAGGTGATAAAT

EPD83

AATCAGATATCGTGAAAGAAGAGAGAGAAAAAGAGAGATGGAAAAGAGAAGAAGAAAGAGCGGCGGTAGGGAATTTTTTTTTTTTTTTTTTTTTTTTTTAGATTTAGGTATCGACAAGTTCTGATATTATGTATGAAATGAGGTTCAATAGTTTATTATATAGATGTTTGTATAATATATATAGTGATTACAAGAGTAATTAGGTATTTATAATTTATAATTATATTTTTAGTTATTTTTTTCTTACAGGTTGATGCTATTAGTGATCTGTTATCGGTGTGTATCTAAATTTCAGAATTGTTATTGATGATTATGTTACAATATATCTGTCTGATTACTGTTTTCTTAATGCTGTAGGTGACAAAT

EPD85

AATTAGATATCGTGAAAGAAGAGAGAGAAAAAGAGAGATGGAAAAGAGATGAAGAAAGAGCGGCGGTAGGGAATTTTTTTTTTTTTTTTTTTTTTTTTAGATTTAGGTATCGACAAGTTCTGATATTATGTATGAAATGAGGTTCAATAGTTTATTATATAGATGTTTGTATAATATATATAGTGATTACAAGAGTAATTAGGTATCTATAATTTACAATTATATTTTTAGTTATTTTTTTCTTACAGGTTGATGCTATTAGTGATCTGTTACCGGTGTGTATCTAAATTTCAGAATTGTTATTGATGATTATGTTATAATATATCTGTCTGATTACTGTTTTTTTAATGTTGCAGGTGATAAAT

***cmt3*- EK5’LTR-CMT1**

EPC86

AATTAGATATTGTGAAGGAAGAGAGAGAAAAAGAGAGATGGAAAAGAGAAGAAGAAAGAGTGGTGGTAGGGAATTTTTTTTTTTTTTTTTTTTTTTTTAGATTTAGGTATTGATAAGTTTTGATATTATGTATGAAATGAGGTTTAACAGTTTATTATATAGATGTTTGTATAATATATATAGTGATTATAAGAGTAATTAGGTATTTATAATTTATAATTATATTTTTAGTTATTTTTTCTTTATAGGTTGATGTTATTAGTGACTTGTTATTGGTGTGTATTTAAATTTTAGAATTGTTATTGATGATTATGTTATAATATATTTGTTTGATTATTGTTTTTTTAATGTTGTAGGTGAAAAT

EPC87

AATTAGATATTGTGAAAGAAGAGAGAGAAAAAGAGAGATGGAAAAGAGAAGAAGAAAGAGTGGTGGTAGGGAATTTTTTTTTTTTTTTTTTTTTTTTTTAGATTTAGGTATTGATAAGTTTTGATATTATGTATGAAATGAGGTTTAATAGTTTATTATATAGATGTTTGTATAATATATATAGTGATTATAAGAGTAATTAGGTATTTATAATTTATAATTATATTTTTAGTTATTTTTTTTTTATAGGTTGATGTTATTAGTGATTTGTTATTGGTGTGTATTTAAATTTTAGAATTGTTATTGATGATTATGTTATAATATATTTGTTTGATTATTGTTTTTTTAATGTTGTAGGTGATAAAT

EPC90

AATTAGATATTGTGAAAGAAGAGAGAGAAAAAGAGAGATGGAAAAGAGAAGAAGAAAGAGTGGTAGTAGGGAATTTTTTTTTTTTTTTTTTTTTAGATTTAGGTATTGATAAGTTTTGATATTATGTATGAAATGAGGTTTAATAGTTTATTATATAGATGTTTGTATAATATATATAGTGATTATAAGAGTAATTAGGTATTTATAATTTATAATTATATTTTTAGTTATTTTTTTTTTATAGGTTGATGTTATTAGTGATTTGTTATTGGTGTGTATTTAAATTTTAGAATTGTTATTGATGATTATGTTATAATATATTTGTTTGATTATTGTTTTTTTAATGTTGTAGGTGATAAAT

EPC91

AATTAGATATTGTGAAAGAAGAGAGAGAAAAAGAGAGATGGAAAAGAGAAGAAGAAAGAGTGGTGGTAGGGAATTTTTTTTTTTTTTTTTTTTTTAGATTTAGGTATTGATAAGTTTTGATATTATGTATGAAATGAGGTTTAATAGTTTATTATATAGATGTTTGTATAATATATATAGTGATTATAAGAGTAATTAGGTATTTATAATTTATAATTATATTTTTAGTTATTTTTTTTTATAGGTTGATGTTATTAGTGATTTGTTATTGGTGTGTATTTAAATTTTAGAATTGTTATTGATGATTATGTTATAATATATTTGTTTGATTATTGTTTTTTTAATGTTGTAGGTGATAAAT

EPC93

AATTAGATATTGTGAAAGAAGAGAGAGAAAAAGAGAGATGGAAAAGAGAAGAAGAAAGAGTGGTGGTAGGGAATTTTTTTTTTTTTTTTTTTTTTTTAGATTTAGGTATTGATAAGTTTTGATATTATGTATGAAATGAGGTTTAATAGTTTATTATATAGATGTTTGTATAATATATATAGTGATTATAAGAGTAATTAGGTATTTATAATTTATAATTATATTTTTAGTTATTTTTTTTTTATAGGTTGATGTTATTAGTGATTTGTTATTGGTGTGTATTTAAATTTTAGAATTGTTATTGATGATTATGTTATAATATATTTGTTTGATTATTGTTTTTTTAATGTTGTAGGTGATAAAT

EPC94

AATTAGATATTGTGAAAGAAGAGAGAGAAAAAGAGAGATGGAAAAGAGAAGAAGAAAGAGTGGTGGTAGGGAATTTTTTTTTTTTTTTTTTTTTTTTTTTAGATTTAGGTATTGATAAGTTTTGATATTATGTATGAAATGAGGTTTAATAGTTTATTATATAGATGTTTGTATAATATATATAGTGATTATAAGAGTAATTAGGTATTTATAATTTATAATTATATTTTTAGTTATTTTTTTTTTATAGGTTGATGTTATTAGTGATTTGTTATTGGTGTGTATTTAAATTTTAGGATTGTTATTGATGATTATGTTATAATATATTTGTTTGATTATTGTTTTTTTAATGTTGTAGGTGATAAGT

EPC95

AATTAGATATTGTGAAAGAAGAGAGAGAAAAAGAGAGATGGAAAAGAGAAGAAGAAAGAGTGGTGGTAGGGAATTTTTTTTTTTTTTTTTTTTTTTTTAGATTTAGGTATTGATAAGTTTTGATATTATGTATGAAATGAGGTTTAATAGTTTATTATATAGATGTTTGTATAATATATATAGTGATTATAAGAGTAATTAGGTATTTATAATTTATAATTATATTTTTAGTTATTTTTTTTTTATAGGTTGATGTTATTAGTGATTTGTTATTGGTGTGTATTTAAATTTTAGAATTGTTATTGATGATTATGTTATAATATATTTGTTTGATTATTGTTTTTTTAATGTTGTAGGTGATAAAT

EPC96

AATTAGATATTGTGAAAGAAGAGAGAGAAAAAGAGAGATGGAAAAGAGAAGAAGAGAGAGTGGTGGTAGGGAATTTTTTTTTTTTTTTTTTTTTTTTAGATTTAGGTATTGATAAGTTTTGATATTATGTATGAAATGAGGTTTAATAGTTTATTATATAGATGTTTGTATAATATATATAGTGATTATAAGAGTAATTAGGTATTTATAATTTATAATTATATTTTTAGTTATTTTTTTTTTATAGGTTGATGTTATTAGTGATTTGTTATTGGTGTGTATTTAAATTTTAGAATTGTTATTGATGATTATGTTATAATATATTTGTTTGATTATTGTTTTTTTAATGTTGTAGGTGATAAAT

EPC97

AATTAGATATTGTGAAAGAAGAGAGAGAAAAAGAGAGATGGAAAAGAGAAGAAGAAAGAGTGGTGGTAGGGAATTTTTTTTTTTTTTTTTTCCTTTTTAAAATTAAGGATTGATAAGTTTTGAAATTATGGATGAAATGAGGGTTAATAATTTATTATATAAATGTTTGGATAAAATATATAGTGATTATAAAAATAATTAAGTATTTATAATTTATAATTATATTTTTAGTTATTTTTTTTTATAGGTGGAGGTTATTATTGAGTTGTTATTGGGGTGTATTTAAATTTTAGAAATGTGATTGAGGATAATGTTATAATATATTTGTGTGAGAATAGTGTTTTTAATGTGGTAGGTGAGAAAT

EPC99

AATTAGATATTGTGAAAGAAGAGAGAGAAAAAGGGAGATGGAAAAGAGAAGAAGAAAGAGTGGTGGTAGGGAATTTTTTTTTTTTTTTTTTTTTTTTAGATTTAGGTATTGATAAGTTTTGATATTATGTATGAAATGAGGTTTAATAGTTTATTATATAGATGTTTGTATAATATATATAGTGATTATAAGAGTAATTAGGTATTTATAATTTATAATTATATTTTTAGTTATTTTTTTTTTATAGGTTGATGTTATTAGTGATTTGTTATTGGTGTGTATTTAAATTTTAGAATTGTTATTGATGATTATGTTATAATATATTTGTTTGATTATTGTTTTTTTAATGTTGTAGGTGATAAAT

***kyp2*-EK5’LTR-CMT1**

KEP50

ATTGTGAAAGAAGAGAGAGAAAAAGAGAGATGGAAAAGGGAAGAAGAAAGAGTGGTGGTAGGGAATTTTTTTTTTTTTTTTTTTTTTAGATTTAGGTATTGATAAGTTTTGATATTATGTATGAAATGAGGTTTAATAGTTTATTATATAGATGTTTGTATAATATATATAGTGATTATAAGAGTAATTAGGTATTTATAATTTATAATTATATTTTTAGTTATTTTTTTTTTATAGGTTGATGTTATTAGTGATTTGTTATTGGTGTGTATTTAAATTTTAGAATTGTTATTGATGATTATGTTATAATATATTTGTTTGATTATTGTTTTTTTAATGTTGTAGGTGATAAAT

KEP51

ATTGTGAAAGAAGAGAGAGAAAAAGAGAGATGGAAAAGAGAAGAAGAAAGAGTGGTGGTAGGGAATTTTTTTTTTTTTTTTTTTTTTTTTAGATTTAGGTATTGATAAGTTTTGATATTATGTATGAAATGAGGTTTAATAGTTTATTATATAGATGTTTGTATAATATATATAGTGATTATAAGAGTAATTAGGTATTTATAATTTATAATTATATTTTTAGTTATTTTTTTTTTTATAGGTTGATGTTATTAGTGATTTGTTATTGGTGTGTATTTAAATTTTAGAATTGTTATTGATGATTATGTTATAATATATTTGTTTGATTATTGTTTTTTTAATGTTGTAGGTGATAAAT

KEP53

ATTGTGAAAGAAGAGAGAGAAAAAGAGAGATGGAAAAGAGAAGAAGAAAGAGTGGTGGTAGGGAATTTTTTTTTTTTTTTTTTTTTTTTTAGATTTAGGTATTGATAAGTTTTGATATTATGTATGAAATGAGGTTTAATAGTTTATTATATAGATGTTTGTATAATATATATAGTGATTATAAGAGTAATTAGGTATTTATAATTTATAATTATATTTTTAGTTATTTTTTTTTTATAGGTTGATGTTATTAGTGATTTGTTATTGGTGTGTATTTAAATTTTAGAATTGTTATTGATGATTATGTTGTAATATATTTGTTTGATTATTGTTTTTTTAATGTTGTAGGTGATAAAT

KEP54

ATTGTGAAAGAAGAGAGAGAAAAAGAGAGATGGAAAAGAGAAGAAGAAAGAGTGGTGGTAGGGAATTTTTTTTTTTTTTTTTTTTTTTTAGATTTAGGTATTGATAAGTTTTGATATTATGTATGAAATGAGGTTTAATAGTTTATTATATAGATGTTTGTATAATATATATAGTGATTATAAGAGTAATTAGGTATTTATAATTTATAATTATATTTTTAGTTATTTTTTTTTTATAGGTTGATGTTATTAGTGATTTGTTATTGGTGTGTATTTAAATTTTAGAATTGTTATTGATGATTATGTTATAATATATTTGTTTGATTATTGTTTTTTTAATGTTGTAGGTGATAAAT

KEP59

AAAGAGACGGGTCAACAAAGAGGGAGAAAAAGAGAGATGGAAAAGAGAAGAAGAAAGAGTGGGGGGGGGGAATTTTTTTTTTTTTTTTTTTTTTTTTAGATTTAGGTATTGATAAGTTTTGATATTATGTATGAAATGAGGTTTAATAGTTTATTATATAGATGTTTGTATAATATATATAGTGATTATAAGAGTAATTAGGTATTTATAATTTATAATTATATTTTTAGTTATTTTTTTTTTATAGGTTGATGTTATTAGTGATTTGTTATTGGTGTGTATTTAAATTTTAGAATTGTTATTGATGATTATGTTATAATATATTTGTTTGATTATTGTTTTTTTAATGTTGTAGGTGATAAAT

KEP61

GTGAGAGAAAAAGAGAGATGGAAAAGAGAAGAAGAAAGAGTGGGGGTAGGGAATTTTTTTTTTTTTTTTTTTTTTTTTTAGATTTAGGTATTGATAAGTTTTGATATTATGTATGAAATGAGGTTTAATAGTTTATTATATAGATGTTTGTATAATATATATAGTGATTATAAGAGTAATTAGGTATTTATAATTTATAATTATATTTTTAGTTATTTTTTTTTTATAGGTTGATGTTATTAGTGATTTGTTATTGGTGTGTATTTAAATTTTAGAATTGTTATTGATGATTATGTTATAATATATTTGTTTGATTATTGTTTTTTTAATGTTGTAGGTGATAAAT

KEP7

ATTGTGAAAGAAGAGAGAGAAAAAGAGAGATGGAAAAGAGAAGAAGAAAGAGTGGTGGTAGGGAATTTTTTTTTTTTTTTTTTTTTTTTTTAGATTTAGGTATTGATAAGTTTTGATATTATGTATGAAATGAGGTTTAATAGTTTATTATATAGATGTTTGTATAATATATATAGTGATTATAAGAGTAATTAGGTATTTATAATTTATAATTATATTTTTAGTTATTTTTTTTTTATAGGTTGATGTTATTAGTGATTTGTTATTGGTGTGTATTTAAATTTTAGAATTGTTATTGATGATTATGTTATAATATATTTGTTTGATTATTGTTTTTTTAATGTTGTAGGTGATAAAT

KEP8

ATTGTGAAAGAAGAGAGAGAAAAAGAGAGATGGAAAAGAGAAGAAGAAAGAGTGGTGGTAGGGAATTTTTTTTTTTTTTTTTTTTTTTTTAGATTTAGGTATTGATAAGTTTTGATATTATGTATGAAATGAGGTTTAATAGTTTATTATATAGATGTTTGTATAATATATATAGTGATTATAAGAGTAATTAGGTATTTATAATTTATAATTATATTTTTAGTTATTTTTTTTTTATAGGTTGATGTTATTAGTGATTTGTTATTGGTGTGTATTTAAATTTTAGAATTGTTATTGATGATTATGTTATAATATATTTGTTTGATTATTGTTTTTTTAATGTTGTAGGTGATAAAT

***ago4*- EK5’LTR-CMT1**

AEP22

AATCAGATATCGTGAAAGAAGAGAGAGAAAAAGAGAGATGGAAAAGAGAAGAAGAAAGAGCGGCGGTAGGGAATTTTTTTTTTTTTTTTTTTTTTTAGATTTAGGTATCGATAAGTTCTGATATTATGTATGAAATGAGGTTTAATAGTTTATTATATAGATGTTTGTATAATATATATAGTGATTACAAGAGTAATTAGGTATCTATAATTTACAATTATATTCTTAGTCATTTCTTTCTTACAGGCTGATGCTATTAGTGATCTGTTATCGGTGTGTATTTAAATTTCAGAACTGTTATTGATGATTATGTTGTAATATATCTGTCTGATTACTGTTCTCTTAATGCTGCAGGTGATAAAT

AEP24

AATCAGACATCGTGAAAGAAGAGAGAGAAAAAGAGAGATGGAAAAGAGAAGAAGAAAGAGCGGCGGTAGGGAATTTTTTTTTTTTTTTTTTTTTTTTAGATTTAGGTGTCGACAAGTTCTGATATTATGTATGAAATGAGGTTCAATAGTTTATTATATAGATGTTTGTACAATATATATAGTGATTACAAGAGTAATTAGGTATCTATAATTTACAATTATATTTTTAGTTATTTTTTCCTTACAGGCTGATGCTATTAGTGATCTGTTACCGGTGTGTATCTAAACTTCAGAACTGTCATTGATGATTATGTTACAATATATCTGTCTGATTACTGTTCTCTTAATGCTGCAGGTGACAAAT

AEP25

AATCAGATATCGTGAAAGAAGAGAGAGAAAAAGAGAGATGGAAAAGAGAAGAAGAAAGAGCGGCGGTAGGGAATTTTTTTTTTTTTTTTTTTCTTTTTTAGATTTAGGTATCGACAAGTTCTGATATTATGTATGAAATGAGGTTCAATAGTTTATTATATAGATGTTTGTATAATATATATAGTGATTACAAGAGTAATTAGGTATTTATAATTTACAATTATATTTTTAGTTATTTTTTTCTTACAGGCTGATGTTATTAGTGATCTGTTACCGGTGTGTATCTAAATTTCAGAACTGTCATTGATGATTATGTTATAATATATCTGTCTGATCACTGTTCTTTTAATGCTGTAGGTGACAAAT

AEP26

AATCAGATATCGTGAAAGAAGAGAGAGAAAAAGAGAGATGGAAAAGGGAAGAAGAAAGAGCGGCGGTAGGGAATTTTTTTTTTTTTTTTTTTTTTTTTAAATTTAGGTATCGACAAGTTCTGATATTATGTATGAAATGAGGTTCAATAGTTTATTATATAGATGTTTGTACAATATATATAGTGATTATAAAAGTAATTAGGTATTTATAATTTATAATTATATTTTTATTTCTTTCTTCTTTATAGGCCGAATTTTTTATTTCTCCGTTCCCGGCGTGTATCTATATTTCCGAA

AEP27

AATCAGATATCGTGAAAGAAGAGAGAGAAAAAGAGAGATGGAAAAGAGAAGAAGAAAGAGCGGCGGTAGGGAATTTTTTTTTTTTTTTTTTTCTTTAGATTTAGGTATCGACAAGTTCTGATATTATGTATGAAATGAGGTTCAATAGTTTATTATATAGATGTTTGTATAATATATATAGTGACTATAAGAGTAATTAGGTATTTATAATTTATAATTATATTTTTAGTTATTTTTTTTTTACAGGCTGATGCTATTAGTGATCTGTTACCGGTGTGTATCTAAATTTTAGAATTGCTATTGATGATTATGTTATAATATATCTGTCTGATCATTGCTTTCTTAATGCTGCAGGTGACAAAT

AEP28

AATCAGATATCGTGAAAGAAGAGAGAGAAAAAGAGAGATGGAAAAGAGAAGAAGAAAGAGCGGCGGTAGGGAATTTTTTTTTTTTTTTTTTTCTTTAGATTTAGGTATCGATAAGTTCTGATATTATGTATGAAATGAGGTTCAATAGTTTATTATATAGATGTTTGTACAATATATGTAGTGATTACAAGAGTAATTAGGTATCTATAATTTACAATTATATTTTTAGTTATTTTTTCCTTACAGGCTGATGCTATTAGTGATCTGTTACCGGTGTGTATCTAAACTTCAGAACTGTCATTGATGATTATGTTACAATATATCTGTCTGATTACTGTTCTCTTAATGCTGCAGGTGACAAAT

AEP29

AATCAGATATCGTGAAAGAAGAGAGAGAAAAAGAGAGATGGAAAAGAGAAGAAGAAAGAGCGGCGGTAGGGAATTTTTTTTTTTTTTTTTTTTTTTTTAGATTTAGGTATCGACAAGTTCTGATATTATGTATGAAATGAGGTTCAATAGTTTATTATATAGATGTTTGTATAATATATATAGTGATTACAAGAGTAATTAGGTATTTATAATTTACAATTATATTTTTAGTTATTTCTTTCTTACAGGCTGATGCTATTAGTGATCTGTTACCGGTGTGTATTTAAATTTTAGAACTGTTATTGATGATTATGTTATAATATATCTGTCTGATTACTGCTCTCTTAATGCTGCAGGTGACAAAT

AEP30

AATCAGACATCGTGAAAGAAGAGAGAGAAAAAGAGAGATGGAAAAGAGAAGAAGAAAGAGCGGCGGTAGGGAATTTTTTTTTTTTTTTTTTTTTTTATATTTAGGTGTCGACAAGTTCTGAGATTATGTATGAAATGAGGCGCAATAGTTTATTACATAGATGTTTGTGCAATATATATAGTGAGTACAAGAGAAATTATGTATCTATAATTTACAATTATATTCTTAGTTATTTCTTTCTTACAGGCTGATGCGATTAGTGATCTGTCACCGGGGTGTATCTAAATTTCAGAACTGTGATTGATGATTATGTTACAATATATCTGTCTGATTACTGCTCTCTTAATGCTGTAGGTGACAAAT

AEP33

AATCAGACATCGTGAAAGAAGAGAGAGAAAAAGAGAGATGGAAAAGAGAAGAAGAAAGAGCGGCGGTAGGGAATTTTTTTTTTTTTTTTTTTTTTTTAGATTTAGGTATCGATAAGTTTTGGTATTATGTATGAAATGAGGTTTAATAGTTTATTATATAGATGTTTGTATAATATATATAGTGATTACAAGAGTAATTAGGTATCTATAATTTACAATTATATTTTTAGTTATTTTTTTCTTACAGGCTGATGCTATTAGTGATCTGTTATCGGTGTGTATTTAAATTTTAGAATTGTTATTGATGATTATGTTATAATATATTTGTTTGATTATTGTTTTTTTAATGTTGTAGGTGATAAAT

AEP34

AATCAGATATCGTGAAAGAAGAGAGAGAAAAAGAGAGATGGAAAAGAGAAGAAGAAAGAGCGGCGGTAGGGAATTTTTTTTTCTTTTTTTTTTTTTTAGATTTAGGTATCGACAAGCTCTGATATTATATATGAAATGAGGTTCAATAGTTTATTGTATAGATGTTTGTATAATATATATAGTGATTATAAGAGTAATTAGGTATCTATAATTTACAATTATATTTTTAGTCATTTTTTTTTTACAGGTTGATGCTATTAGTGATCTGTTATCGGTGTGTATCTAAATTTTAGAGCTGTTATTGATGATTATGTTACAATATATCTGTCTGATTACTGTTTTTTTAATGCTGTAGGTGATAAAT

***hen1*- EK5’LTR-CMT1**

HEP75

AATTAGATATCGTGAAAGAAGAGAGAGAAAAAGAGAGATGGAAAAGAGAAGAAGAAAGAGCGGCGGTAGGGAATTTTTTTTTTTTTTTTTTTTTTTAGATTTAGGTGTCGATAAGTTCTGATATTATGTATGAAATGAGGTTTAATAGTTTATTATATAGATGTTTGTATAATATATATAGTGATTACAAGAGTAATTAGGTATCTATAATTTATAATTATATTTTTAGTTATTTTTTTCTTACAGGCTGATGCTATTAGTGATCTGTTACCGGTGTGTATCTAAATTTCAGAACTGTTATTGATGATTATGTTACAATATATCTGTCTGATTACTGTTCTCTTAATGCTGCAGGTGATAAAT

HEP76

AATCAGACATCGTGAAAGAAGAGAGAGAAAAAGAGAGATGGAAAAGAGAAGAAGAAAGAGCGGCGGTAGGGAATTTTTTTTTTTTTTTTTTTTTTTTAGATTTAGGTATCGACAAGTTCTGATATTATGTATGAAATGAGGCTCAATAGTTTATTATATAGATGTTTGTATAATATATATAGTGATTACAAGAGTAATTAGGTATCTATAATTTACAATTATATTTTTAGTTATTTCTTTCTTACAGGCTGATGTTATTAGTGATCTGTTATCGGTGTGTATTTAAATTTCAGAACTGTTATTGATGATTATGTTATAATATATCTGTCTGATTACTGTTTTCTTAATGCTGCAGGTGATAAAT

HEP77

AATCAGATATCGTGAAAGAAGAGAGAGAAAAAGAGAGATGGAAAAGAGAAGAAGAAAGAGCGGCGGTAGGGAATTTTTTTTTTTTTTTTTTTTTTTAGATTTAGGTATCGACAAGTTCTGATATTATGTATGAAATGAGGTTTAATAGTTTATTATATAGATGTTTGTATAATATATATAGTGATTACAAGAGTAATTAGGTATCTATAATTTACAATTATATTTTTAGTTATTTTTTTCTTACAGGCTGATGCTATTAGTGATCTGTTATCGGTGTGTATTTAAATTTCAGAACTGTTATTGATGATTATGTTATAATATATCTGTCTGATTACTGTTTTTTTAATGCTGCAGGTGATAAAT

HEP78

AATCAGACATCGTGAAAGAAGAGAGAGAAAAAGAGAGATGGAAAAGAGAAGAAGAAAGAGCGGCGGTAGGGAATTTTTTTTTTTTTTTTTTTAGATTTAGGTATCGACAAGTTCTGATATTATGTATGAAATGAGGCTCAATAGTTTATTATATAGATGTTTGTACAATATATATAGTGATTACAAGAGTAATTAGGTATTTATAATTTATAATTATATTTTTAGTTATTTTTTTTTTACAGGTTGATGCTATTAGTGATCTGTTACCGGTGTGTATCTAAATTTCAGAACTGTCATTGATGATTATGTTACAATATATCTGTCTGATTACTGCTCTCTTAATGCTGCAGGTGACAAAT

HEP80

AATCAGATGTCGTGAAAGAAGAGAGAGAAAAAGAGAGATGGAAAAGAGAAGAAGAAAGAGCGGCGGTAGGGAATTTTTTTTTTTTTTTTTTTTTTTAGATTTAGGTATCGACAAGTTCTGATATTATGTATGAAATGAGGTTCAATAGTTTATTATGTAGATGTTTGTACAATATATATAGTGATTACAAGAGTAATTAGGTATTTATAATTTATAATTATATTTTTAGTTATTTTTTTTTTACAGGTTGATGCTATTAGTGATCTGTTACCGGTGTGTATCTAAATTTCAGAACTGTCATTGATGATTATGTTACAATATATCTGTTTGATTATTGTTTTTTTAATGCTGCAGGTGACAAAT

HEP81

AATCAGACATCGTGAAAGAAGAGAGAGAAAAAGAGAGATGGAAAAGAGAAGAAGAAAGAGCGGCGGTAGGGAATTTTTTTTTTTTTTTTTTTTTTTCTTTAGATTTAGGTATCGACAAGTTCTGATATTATGTATGAAATGAGGTTCAATAGTTTATTATATAGATGTTTGTACAATATATATAGTGATTACAAGAGTAATTAGGTATTTATAATTTATAATTATATTCTTAGTTATTTCTTCCTTACAGGCTGATGCTATTAGTGATCTGTCACCGGTATGTATCTAAATTTCAGAACTGTTATTGATGATTATGTTACAATATATCTGTCTGATTACTGTTCTTTTAATGCTGCAGGTGATAAAT

HEP82

AATCAGATATCGTGAAAGAAGCGAGAGAAAAAGAGAGATGGAGAAGAGAAGAAGAAAGAGCGGCGGTAGGGAATTTTTTTTTTTTTTTTTTTTTCTTTAGATTTAGGTATCGATAAGTTCTGATATTATGTATGAAATGAGGTTCAATAGTTTATTATATAGATGTTTGTACAATATATATAGTGATTACAAGAGTAATTAGGTATCTATAATTTACAATTATATTCTTAGTTATTTCTTTTTTACAGGCTGATGCTATTAGTGATCTGTTACCGGTGTGTATCTAAATTTCAGAATTGTTATTGATGATTATGTTACAATATATCTGTCTGATTACTGTTCTTTTAATGCTGCAGGTGATAAAT

HEP84

AATTAGATATCGTGAAAGAAGAGAGAGAAAAAGAGAGATGGAAAAGAGAGGAAGAAAGAGTGGCGGTAGGGAATTTTTTTTTTTTTTTTTTTTTTTTAGATTTAGGTATCGACAAGTTCTGATATTATGTATGAAATGAGGTTCAATAGTTTATTGTATAGATGTTTGTATAATATATATAGTGATTATAAGAGTAATTAGGTATTTATAATTTATAATTATATTTTTAGTTATTTTTTTCTTACAGGTTGATGCTATTAGTGATCTGTTACCGGTGTGTATTTAAATTTCAGAACTGTCATTGATGATTATGTTACAATATATCTGTCTGATTACTGTTTTCTTAATGCTGCAGGTGACAAAT

HEP85

AATCAGATATCGTGAAAGAAGAGAGAGAAAAAGAGAGATGGAAAAGAGAAGAAGAAAGAGCGGCGGTAGGGAATTTTTTTTTTTTTTTTTTTTTTTTTAAATTTAGGTATCGATAAGTTCTGATATTATGTATGAAATGAGGTTTAATAGTTTATTATATAGATGTTTGTATAATATATATAGTGATTATAAGAGTAATTAGGTATTTATAATTTATAATTATATTTTTAGTTATTTTTTTCTTACAGGTTGATGCTATTAGTGATTTGTTATCGGTGTGTATTTAAATTTCAGAATTGTTATTGATGATTATGTTATAATATATCTGTCTGATTACTGTTTTCTTAATGCTGCAGGTGATAAAT

HEP86

AATCAGATATCGTGAAAGAAGAGAGAGAAAAAGAGAGATGGAAAAGAGAAGAAGAAAGAGCGGCGGTAGGGAATTTTTTTTTTTTTTTTTTTTTTTTTAGATTTAGGTATTGATAAGTTCTGATATTATGTATGAAATGAGGTTTAATAGTTTATTATATAGATGTTTGTATAATATATATAGTGATTATAAGAGTAATTAGGTATTTATAATTTATAATTATATTTTTAGTTATTTCTTTCTTACAGGCTGATGTTATTAGTGATCTGTTATCGGTGTGTATTTAAATTTTAGAATTGTTATTGATGATTATGTTACAATATATCTGTCTGATTACTGCTCTCTTAATGCTGCAGGTGACAAAT

***rdr2*- EK5’LTR-CMT1**

REP1

AATCAGATATCGTGAAAGAAGAGAGAGAAAAAGAGAGATGGAAAAGAGAAGAAGAAAGAGCGGCGGTAGGGAATTTTTTTTTTTTTTTTTTTTTAGATTTAGGTATCGACAAGTTCTGATATTATGTATGAAATGAGGTTCAATAGTTTATTGTATAGATGTTTGTACAATATATATAATGATTATAAGAGTAATTAGGTATTTATAATTTATAATTATATTTTTAGTTATTTTTTTCTTACAGGCTGATGCTATTAGTGATCTGTTACCGGTGTGTATCTAAATTTCAGAACTGTCATTGATGATTATGTTACAATATATCTGTCTGATTACTGTTCTCTTAATGCTGCAGGTGACAAAT

REP2

AATTAGATATCGTGAAAGAAGAGAGAGAAAAAGAGAGATGGAAAAGAGAAGAAGAAAGAGCGGCGGTAGGGAATTTTTTTTTTTTTTTTTTTTTTTTTAGATTTAGGTATCGATAAGTTCTGATATTATGTATGAAATGAGGTTTAATAGTTTATTATATAGATGTTTGTATAATATATATAGTGATTATAAGAGTAATTAGGTATCTATAATTTACAATTATATCCTTAGTTATTTTTTTCTTACAGGCTGATGCTATTAGTGATCTGTTATCGGTGTGTATTTAAATTTCAGAACTGTTATTGATGATTATGTTACAATATATCTGTCTGATTACTGTTCTCTTAATGCTGCAGGTGATAAAT

REP3

AATCAGATATCGTGAAAGAAGAGAGAGAAAAAGAGAGATGGAAAAGAGAAGAAGAAAGAGCGGCGGTAGGGAATTTTTTTTTTTTTTTTTTTTTTTAGATTTAGGTATCGACAAGTTTTGATATTATGTATGAAATGAGGTTCAATAGTTTATTATATAGATGTTTGTACAATATATATAGTGATTACAAGAGTAATTAGGTATCTATAATTTACAATTATATTCTTAGTTATTTTTTTCTTACAGGCTGATGCTATTAGTGATCTGTTACCGGTGTGTATTTAAATTTCAGAACTGTTATTGATGATTATGTTATAATATATCTGTCTGATTACTGTTCTCTTAATGCTGCAGGTGACAAAT

REP5

AATCAGATATCGTGAAAGAAGGGAGAGAAAAAGAGAGATGGAAAAGAGAAGAAGAAAGAGCGGCGGTAGGGAATTTTTTTTTTTTTTTTTTTTTTTAGATTTAGGTATCGATAAGTTTTGATATTATGTATGAAATGAGGTTCAATAGTTTATTATATAGATGTTTGTACAATATATATAGTGATTACAAGAGTAATTAGGTATCTATAATTTATAATTATATTTTTAGTTATTTTTTTCTTACAGGCTGATGCTATTAGTGATCTGTCACCGGTGTGTATCTAAATTTCAGAACTGTTATTGATGATTATGTTACAATATATCTGTCTGATTACTGCTCTCTTAATGCTGCAGGTGACAAAT

REP12

AATCAGATATCGTGAAAGAAGAGAGAGAAAAAGAGAGATGGAAAAGAGAAGAAGAAAGAGCGGCGGTAGGGAATTTTTTTTTTTTTTTTTTTTTTTTAGATTTAGGTATCGACAAGTTCTGATATTATGTATGAAATGAGGTTTAATAGTTTATTATATAGATGTTTGTACAATATATATAGTGATTATAAGAGTAATTAGGTACCTATAATTTATAATTATATCTTTAGTCATTTCTTTCTTACAGGCTGATGCTATTAGTGATCTGTCATCGGTGTGTATCTAAATTTTAGAACTGTTATTGATGATTATGTTATAATATATTTGTCTGATTATTGTTCTCTTAATGTTGCAGGTGACAAAT

REP13

AATCAGATATCGTGAAAGAAGAGAGAGAAAAAGAGAGATGGAAAAGAGAAGAAGAAAGAGCGGCGGTAGGGAATTTTTTTTTTTTTTTTTTTTTTAGATTTAGGTATCGACAAGTTCTGATATTATGTATGAAATGAGGTTTAATAGTTTATTATATAGATGTTTGTACAATATATATAGTGATTATAAGAGTAATTAGGTATCTATAATTTATAATTATATTCTTAGTTATTTTTTTCTTACAGGCTGATGTTATTAGTGATCTGTCATCGGTGTGTATCTAAATTTCAGAACTGTTATTGATGATTATGTTATAATATATCTGTCTGATTACTGTTCTCTTAATGCTGCAGGTGATAAAT

REP14

TTCGCACATAGCGCGAAAAAAGAGAGAGAAAAGAGAGAGGGAAAAGAGAAGAAGAAAGAGCGGCGGTGGGGAATTTTTTTTTTTTTTTTTTTTCTTTAAATTTAGGTACCCACAAGTTCTGATATTATGTATGAAACGAGGTTTTATATTTTATTATATAGATGTTTGTACAAAATATATATTGATTATACAAGTAATTAGGTATTTATAATTTAAAATTATATTCTTACTTATTTTTTTCTTATAGGCGGATGCTATTACTGATCTGTTACCGGTGTGTATTTAAATTTTAAAACTGTTATTGATGATTATGTTATAATATATCTGTCTGATTATTGTTCCCCTAATGCTGCAGGTGATAACT

REP15

AATTATATATTGTGAAAAGAAGAGAGAGAAAAAGAGAGATGGAAAATAGAAAAAAAAAGAACGGCGGTTTGGAATTTTTTTTTTTTTTTTTTTTTTTTAAATTTACGTATCAACAATTTCTTTATATTATGTATGAAATGAGGTTCAATATTTTATTATATTCATGTTTATTTAAAAAATATAAAGATTACAAAAATAATTAAGTATTTATAATTTATAATTATATTTTTATTCATCTTTTTTTTACAGGCTGACGCTATTAATGATTTGATATCGGCTTCTATCTAAATTTCCCAATTGTTCTCTATTACTATGTTTCAT

REP16 TTTTTTTAGATTTAGGTATCGACAAGTTCTGATATTATGTATGAAATGAGGTTCAATAGTTTATTATATAGATGTTTGTACAATATATATAGTGATTATAAGAGTAATTAGGTATTTATAATTTATAATTATATTCTTAGTTATTTTTTTCTTACAGGCTGATGTTATTAGTGATCTGTCATCGGTGTGTATCTAAATTTCAGAACTGTTATTGATGATTATGTTATAATATATCTGTCTGATTACTGTTCTCTTAATGCTGCAGGTGATAAAT

REP99

AATCAGGTATCGTGAAAGAAGAGAGAGAAAAAGAGAGATGGAAAAGAGAAGAAGAAAGAGCGGCGGTAGGGAATTTTTTTTTTTTTTTTTTTTTCTTTAGATTTAGGTATCGACAAGCTCTGATATTATGTATGAAATGAGGTTTAATAGTTTATTATATAGATGTTTGTATAATATATATAGTGATTATAAGAGTAATTAGGTATCTATAATTTATAATTATATTTTTAGTTATTTTTTTCTTACAGGCTGATGTTATTAGTGATCTGCTACCGGTGTGTATTTAAATTTTAGAACTGTTATTGATGATTATGTTATAATATATCTGTCTGATTACTGTTTTTTTAATGCTGCAGGTGATAAAT

**Part-II: EK coding region (EKcr)**

**Sequence used for methylation analysis** (primers-underlined)

GTGGGGAATGTTGTGGATTTGGGCTATTTGGTTGTGGGCTTGAGGAATGTGGGCTTGTGGATTGTGGGCTTTGTGGCTGTGGGCGTTGTGGCTGTGGGCTGAGTGGAGGAGAAGAACCAGTTGGGCCTGTTTCTGAATTGTGGCCCACAAGAGATTCTTCACTCAACGAAGGAGACGTGATCGGCGAAGTTGGAGACAATGACAGTGATGGTTGTAACGGAGCCGGTGGCGAGAGATTTTCGGACTGCGACGGTGACCGGTGAGGAGCTGAGCACGAGGGCGACGAAGGCGGAGCTGTAGTGAGTGGACGAGCAAGAAGCGGGACTGAGACGGGTCTTGTGTCTTGTGAGAGAGGAGGATCTGATGGAGGTGTGACAGAAGG

**WTLer - EKcr**

LEG1

GTTTTGTGGCTGTGGGCGTTGTGGCTGTGGGTTGAGTGGAGGAGAAGAATCAGTTGGGTTTGTTTCTGAATTGTGGTTTACAAGAGATTTTTCACTCAACGAAGGAGACGTGATCGGCGAAGTTGGAGATAATGACAGTGATGGTTGTAACGGAGTCGGTGGCGAGAGATTTTCGGACTGCGACGGTGACCGGTGAGGAGTTGAGTACGAGGGCGACGAAGGCGGAGTTGTAGTGAGTGG

LEG3

GTTTTGTGGCTGTGGGCGTTGTGGCTGTGGGCTGAGTGGAGGAGAAGAACCAGTTGGGCCTGTTTTTGAATTGTGGCCCACAAGAGATTCTTCACTCGACGAAGGAGACGTGATCGGTGAAGTTGGAGATAATGACAGTGATGGTTGTAACGGAGTTGGTGGCGAGAGATTTTCGGACTGCGACGGTGATCGGTGAGGAGTTGAGTACGAGGGCGACGAAGGCGGAGTTGTAGTGAGTGG

LEG5

GTTTTGTGGCTGTGGACGTTGTGGCTGTGGGTTGAGTGGAGGAGAAGAATTAGTTGGGTTTGTTTTTGAATTGTGGTTTATAAGAGATTTTTTACTCAACGAAGGAGACGTGATCGGCGAAGTTGGAGATAATGACAGTGATGGTTGTAACGGAGCCGGTGGCGAGAGATTTTTGGATTGCGACGGTGACCGGTGAGGAGCTGAGTACGAGGGCGACGAAGGCGGAGTTGTAGTGAGTGG

LEG8

GTTTTGTGGCTGTGGGCGTTGTGGTTGTGGGTTGAGTGGAGGAGAAGAATTAATTGGGTCTGTTTTTGAATTGTGGTTTACAAGAGATTTTTTACTTAATGAAGGAGACGTGATCGGTGAAGTTGGAGACAATGACAGTGATGGTTGTAACGGAGCCGGTGGCGAGAGATTTTCGGATTGCGACGGTGATCGGTGAGGAGCTGAGTACGAGGGCGACGAAGGCGGAGCTGTAGTGAGTGG

LEG9

GTTTTGTGGCTGTGGGCGTTGTGGCTGTGGGCCGAGTGGAGGAGAAGAATCAGTTGGGTCTGTTTCTGAATTGTGGTTTACAAGAGATTTTTCATTCAACGAAGGAGACGTGATCGGCGAAGTTGGAGACAATGACAGTGATGGTTGTAACGGAGTCGGTGGCGAGAGATTTTCGGACTGCGACGGTGATCGGTGAGGAGTTGAGTACGAGGGCGACGAAGGCGGAGCTGTAGTGAGTGG

LEG10

GTTTTGTGGCTGTGGGCGTTGTGGCTGTGGGCTGAGTGGAGGAGAAGAATCAGTTGGGTCTGTTTTTGAATTGTGGTTTACAAGAGATTTTTTACTCAACGAAGGAGACGTGATCGGCGAAGTTGGAGACAATGACAGTGATGGTTGTAACGGAGTCGGTGGCGAGAGATTTTCGGACTGCGACGGTGACCGGTGAGGAGCTGAGTACGAGGGCGACGAAGGCGGAGTTGTAGTGAGTGG

LEG11

GTTTTGTGGCCGTGGGCGTTGTGGCTGTGGGCTGAGTGGAGGAGAAGAATCAGTTGGGTCTGTTTTTGAATTGTGGTTTACAAGAGATTTTTTACTCAACGAAGGAGACGTGATCGGCGAAGTTGGAGACAATGACAGTGATGGTTGTAACGGAGCCGGTGGCGAGAGATTTTCGGACTGCGACGGTGACCGGTGAGGAGCTGAGTACGAGGGCGACGAAGGCGGAGTTGTAGTGAGTGG

LEG12

GTAAAGGGGTTGTGGGCGTTGTGGTTGTGGGTTGAGTGGAGGAGAAGAATTAGTTGGGTCCGTTTTTGAATTGTGGTTTACAAGAGATTTTTTATTTAACGAAGGAGACGTGATCGGTGAAGTTGGAGATAATGACAGTGATGGTTGTAACGGAGCCGGTGGCGAGAGATTTTTGGACTGCGACGGTGATCGGTGAGGAGCTGAGTACGAGGGCGATGAAGGTGGAGCTGTAGTGAGTGG

LEG13

GTTTTGTGGCTGTGGGCGTTGTGGTTGTGGGCTGAGTGGAGGAGAAGAATCAGTTGGGTTTGTTTTTGAATTGTGGTTTATAAGAGATTTTTTATTTAACGAAGGAGACGTGATCGGCGAAGTTGGAGACAATGACAGTGATGGTTGTAACGGAGTCGGTGGCGAGAGATTTTCGGACTGCGACGGTGATCGGTGAGGAGCTGAGTACGAGGGCGATGAAGGCGGAGTTGTAGTGAGTGG

LEG15

GTTTTGTGGTTGTGGGCGTTGTGGCTGTGGGCTGAGTGGAGGAGAAGAATTAGTTGGGTTTGTTTCTGAATTGTGGTTTACAAGAGATTTTTTACTCAACGAAGGAGACGTGATCGGCGAAGTTGGAGACAATGACAGTGATGGTTGTAACGGAGTCGGTGGCGAGAGATTTTCGGATTGCGACGGTGATCGGTGAGGAGTTGAGTATGAGGGCGACGAAGGCGGAGCTGTAGTGAGTGG

LEG2-7

GGTTTGTGGATTGTGGGTTTTGTGGCTGTGGGCGTTGTGGTTGTGGGTTGAGTGGAGGAGAAGAATTAGTTGAGTCTGTTTCTGAATTGTGGTTTACAAGAGATTTTTCACTCAACGAAGGAGACGTGATCGGTGAAGTTGGAGACGATGATAGTGATGGTTGTAACGGAGTCGGTGGCGAGAGATTTTCGGATTGCGACGGTGATCGGTGAGGAGTTGAGTACGAGGGCGACGAAGGCGGAGTTGTAGTGAGTGGACGAGCAAGAAGCGGGATTGAGACGGGTTCTGTGTTTT

LEG2-8

GGTTTGTGGATTGTGGGTTTTGTGGCTGTGGGCGTCGTGGCTGTGGGTTGAGTGGAGGAGAAGAATCAGTTGGGTCTGTTTTTGAATTGTGGTTCACAAGAGATTTTTCACTCAACGAAGGAGACGTGATCGGCGAAGTTGGAGATAATGACAGTGATGGTTGTAACGGAGTCGGTGGCGAGAGATTTTCGGACTGCGACGGTGACCGGTGAGGAGCTGAGTACGAGGGCGACGAAGGCGGAGCTGTAGTGAGTGGACGAGTAAGAAGCGGGACTGAGATGGGTTTTGTGTCTT

LEG2-10

GGTTTGTGGATTGTGGGTTTTGTGGCTGTGGGCGTTGTGGTTGTGGGTTGAGTGGAGGAGAAGAATTAGTTGGGTCTGTTTCTGAATTGTGGTTTACAAGAGATTTTTTACTCAACGAAGGAGACGTGATCGGCGAAGTTGGAGATAATGATAGTGATGGTTGTAACGGAGCCGGTGGCGAGAGATTTTCGGACTGCGACGGTGATCGGTGAGGAGCTGAGTACGAGGGCGATGAAGGCGGAGTTGTAGTGAGTGGACGAGTAAGAAGCGGGACTGAGACGGGTCTTGTGTCTT

***ddm1*-EKcr**

DEG17 GTTTTGTGGCTGTGGGTGTTGTGGCTGTGGGTTGAGTGGAGGAGAAGAATCAGTTGGGTCTGTTTTTGAATTGTGGTTTACAAGAGATTTTTTATTCAACGAAGGAGACGTGATCGGCGAAGTTGGAGACAATGACAGTGATGGTTGTAACGGAGTCGGTGGCGAGAGATTTTCGGATTGCGACGGTGATCGGTGAGGAGTTGAGTACGAGGGCGACGAAGGCGGAGTTGTAGTGAGTGGACGAGTAAGAAGTGGGACTGAGACGGGTCTTGTGTCTT

DEG18

GTTTTGTGGTTGTGGGCGTTGTGGTTGTGGGTTGAGTGGAGGAGAAGAATTAGTTGGGTTTGTTTTTGAATTGTGGTTTACAAGAGATTTTTTATTTAACGAAGGAGACGTGATCGGCGAAGTTGGAGACAATGACAGTGATGGTTGTAACGGAGTCGGTGGCGAGAGATTTTCGGACTGCGACGGTGATCGGTGAGGAGCTGAGTACGAGGGCGACGAAGGCGGAGTTGTAGTGAGTGGACGAGCAAGAAGCGGGACTGAGACGGGTCTTGTGTCTT

DEG20

GTTTTGTGGCTGTGGGCGTTGTGGTTGTGGGTTGAGTGGAGGAGAAGAATCAGTTGGGTTTGTTTTTGAATTGTGGTTTACAAGAGATTTTTTATTCGACGAAGGAGACGTGATCGGCGAAGTTGGAGATAATGACAGTGATGGTTGTAACGGAGTCGGTGGCGAGAGATTTTCGGACTGCGACGGTGATCGGTGAGGAGCTGAGTACGAGGGCGACGAAGGCGGAGCTGTAGTGAGTGGACGAGCAAGAAGCGGGACTGAGACGGGTCTTGTGTCTT

DEG22

GTTTTGTGGTTGTGGGCGTTGTGGTTGTGGGTTGAGTGGAGGAGAAGAATTAGTTGGGTTTGTTTTTGAATTGTGGTTTACAAGAGATTTTTTATTCAACGAAGGAGACGTGATCGGCGAAGTTGGAGATAATGACAGTGATGGTTGTAACGGAGTCGGTGGCGAGAGATTTTCGGATTGCGACGGTGATCGGTGAGGAGCTGAGTACGAGGGTGATGAAGGCGGAGTTGTAGTGAGTGGATGAGTAAGAAGCGGGATTGAGACGGGTCTTGTGTTTT

DEG24

GTTTTGTGGCTGTGGGCGTTGTGGCTGTGGGCTGAGTGGAGGAGAAGAATTAGTTGGGTCTGTTTTTGAATTGTGGTCCACAAGAGATTTTTCACTCAACGAAGGAGACGTGATCGGCGAAGTTGGAGACAATGACAGTGATGGTTGTAACGGAGCCGGTGGCGAGAGATTTTCGGACTGCGACGGTGACCGGTGAGGAGCTGAGTACGAGGGCGACGAAGGCGGAGCTGTAGTGAGTGGACGAGTAAGAAGTGGGACTGAGACGGGTCCTGTGTCTT

DEG26

GTTTTGTGGTTGTGGGCGTTGTGGTTGTGGGTTGAGTGGAGGAGAAGAATTAGTTGGGTTTGTTTTTGAATTGTGGTTTACAAGAGATTTTTTATTTAACGAAGGAGACGTGATCGGTGAAGTTGGAGATAATGATAGTGATGGTTGTAACGGAGTCGGTGGCGAGAGATTTTCGGACTGTGACGGTGATCGGTGAGGAGCTGAGTACGAGGGCGACGAAGGCGGAGTTGTAGTGAGTGGACGAGTAAGAAGCGGGACTGAGACGGGTTTTGTGTTTT

DEG28

ATTTTGTGGCTGTGGGCGTTGTGGCTGTGGGCTGAGTGGAGGAGAAGAATTAGTTGGGTTTGTTTTTGAATTGTGGTTTACAAGAGATTTTTTACTTAACGAAGGAGACGTGATCGGCGAAGTTGGAGATAATGATAGTGATGGTTGTAACGGAGTCGGTGGCGAGAGATTTTCGGACTGCGACGGTGACCGGTGAGGAGCTGAGTACGAGGGCGACGAAGGCGGAGCTGTAGTGAGTGGACGAGCAAGAAGCGGGATTGAGACGGGTCTTGTGTTTT

DEG29

GTTTTGTGGCTGTGGGCGTTGTGGCTGTGGGCTGAGTGGAGGAGAAGAATCAGTTGGGTCTGTTTTTGAATTGTGGTTTACAAGAGATTTTTCACTTAATGAAGGAGACGTGATCGGCGAAGTTGGAGACAATGACAGTGATGGTTGTAACGGAGTCGGTGGCGAGAGATTTTCGGACTGCGACGGTGACCGGTGAGGAGTTGAGTACGAGGGCGATGAAGGTGGAGCTGTAGTGAGTGGACGAGCAAGAAGCGGGACTGAGACGGGTCTTGTGTTTT

DEG30

GTTTTGTGGTTGTGGGTGTTGTGGTTGTGGGTTGAGTGGAGGAGAAGAATTAGTTGGGTCTGTTTTTGAATTGTGGTTTACAAGAGATTTTTTATTCAACGAAGGAGACGTGATCGGCGAAGTTGGAGATAATGACAGTGATGGTTGTAACGGAGTCGGTGGCGAGAGATTTTCGGACTGTGACGGTGACCGGTGAGGAGCTGAGTACGAGGGCGACGAAGGCGGGGTTGTAGCGAGTGGACGAGTAAGAAGCGGGATTGAGACGGGTTTTGTGTTTT

DEG32

GTTTTGTGGCTGTGGGTGTTGTGGCTGTGGGTTGAGTGGAGGAGAAGAATTAGTTGGGTTTGTTTTTGAATTGTGGTTTACAAGAGATTTTTTATTTAACGAAGGAGACGTGATCGGCGAAGTTGGAGACAATGACAGTGATGGTTGTAACGGAGCCGGTGGCGAGAGATTTTCGGACTGCGACGGTGACCGGTGAGGAGCTGAGTACGAGGGCGACGAAGGTGGAGTTGTAGTGAGTGGACGAGTAAGAAGCGGGACTGAGACGGGTCTTGTGTCTT

***cmt3*-EKcr**

CEG33

GTTTTGTGGTTGTGGGTGTTGTGGTTGTGGGTTGAGTGGAGGAGAAGAATTAGTTGGGTTTGTTTTTGAATTGTGGTTTATAAGAGATTTTTTATTTAATGAAGGAGATGTGATTGGCGAAGTTGGAGATAATGATAGTGATGGTTGTAATGGAGTCGGTGGCGAGAGATTTTCGGATTGCGACGGTGATCGGTGAGGAGTTGAGTACGAGGGTGATGAAGGTGGAGTTGCAGGGATTGGATGAGTAAGAAGGGG

CEG35

GTTTTGTGGTTGTGGGTGTTGTGGTTGTGGGTTGAGTGGAGGAGAAGAATTAGTTGGGTTTGTTTTTGAATTGTGGTTTATAAGAGATTTTTTATTTAATGAAGGAGATGTGATTGGCGAAGTTGGAGATAATGATAGTGATGGTCGCAATGGAGTTCGTGGAGAAATATAACCGCC

CEG37

GTTTTGTGGTTGTGGGTGTTGTGGTTGTGGGTTGAGTGGAGGAGAAGAATTAGTTGGGTTTGTTTTTGAATTGTGGTTTATAAGAGATTTTTTATTTAATGAAGGAGATGTGATTGGTGAAGTTGGAGATAATGATAGTGATGGTTGTAATGGAGTTGGTGGTGAGAGATTTTTGGATTGCCACGGTGATGGGTGAGGAGTTGAGTTTGAGGGTGATGAAGGCAGGGTTCTAGTGTTTGGATGAGTAAAAAGGG

CEG38

GTTTTGTGGTTGTGGGTGTTGTGGTTGTGGGTTGAGTGGAGGAGAAGAATTAGTTGGGTTTGTTTTTGAATTGTGGTTTATAAGAGATTTTTCATTTAACGAGGGAGACGTGATCGGCGAAGTTGGAGATAATGATAGTGATGGTTGTAATGGAGTCGGTGGCGAGAGATTTTCGGATTGCGACGGTGATCGGTGAGGAGTTGAGTACGAGGGCGACGAAGGCGGAGTTGTAGTGAGTGGACGAGTAAGAAGCGGGATTGAGACGGG

CEG41

GTTTTGTGGTTGTGGGTGTTGTGGTTGTGGGTTGAGTGGAGGAGAAGAATTAGTTGGGTTTGTTTTTGAATTGTGGTTTATAAGAGATTTTTTATTTAATGAAGGAGATGTGATTGGTGAAGTTGGAGATAATGATAGTGATGGTTGTAATGGAGTTGGTGGGGAGAGCTTTCTGGATTGTCATGGTTTTTATAGA

CEG43

GTTTTGTGGTTGTGGGTGTTGTGGTTGTGGGTTGAGTGGAGGAGAAGAATTAGTTGGGTTTGTTTTTGAATTGTGGTTTATAAGAGATTTTTTATTTAATGAAAGAGATGTGATTGGTGAAGTTAGACATAATGATAATGATGGCTTAACAGAA

CEG44

GTTTTGTGGTTGTGGGTGTTGTGGTTGTGGGTTGAGTGGAGGAGAAGAATTAGTTGGGTTTGTTTTTGAATTGTGGTTATAAGAGATTTTTTATTTAATGAAGGAGATGTGATTGGTGAAGTTGGAGATAATGATAGTGATGGTTGTAATGGAGTCGGTGGCGAGAGATTTAGGATTGCGACGGTGTTGATGAAGA

CEG45

GTTTTGTGGTTGTGGGTGTTGTGGTTGTGGGTTGAGTGGAGGAGAAGAATTAGTTGGGTTTGTTTTTGAATTGTGGTTTATAAGAGATTTTTTATTTAATGAAGGAGATGTGATCGGTGAAGTTGGAGATAATGA

CEG 47

GTTTTGTGGTTGTGGGTGCTGTGGTTGTGGGTTGAGTGGAGGAGAAGAATTAGTTGGGTTTGTTTTTGAATTGTGGTTTATAAGAGATTTTTTATTTAATGAAGGAGATGTGATTGGCGGAGTTGGAGATAATGATAGTGATGGTTGTAACGGAGTCGGTGGTGAGAGATTTTCGGATTGCGACGGTGATCGGTGAGGAGTTGAGTATGAGGGCGATGAATGTGGAGTTGTAGTGATTGAAAGAGAAAGA

CEG 48

GTTTTGTGGTTGTGGGTGTTGTGGTTGTGGGTTGAGTGGAGGAGAAGAATTAGTTGGGTTTGTTTTTGAATTGTGGTTTATAAGAGATTTTTTATTTAATGAAGGAGATGTGATTGGTGAAGTTGGAGATAATGATAGTGATGGTTGTAATGGAGTTGGTGGTGAGAGATATAGGACCCGACGG

***kyp2*-EKcr**

KEG1

GGTTTGTGGATTGTGGGTTTTGTGGTTGTGGGTGTTGTGGTTGTGGGTTGAGTGGAGGAGAAGAATTAGTTGGGTTTGTTTTTGAATTGTGGTTTATAAGAGATTTTTTATTTAACGAAGGAGACGTGATCGGCGAAGTTGGAGATAATGATAGTGATGGTTGTAACGGAGTCGGTGGCGAGAGATTTTCGGATTGCGACGGTGATCGGTGAGGAGTTGAGTACGAGGGTGACGAAGGTGGAGTTGTAGTGAGTGGACGAGTAAGAAGCGGGATTGAGACGGGTTTTGTGTTTT

KEG4

GGTTTGTGGATTGTGGGTTTTGTGGTTGTGGGCGTTGTGGTTGTGGGTTGAGTGGGGGGGAAGAATTAGTTGGGTTTGTTTTTGAATTGTGGTTTACAAGAGATTTTTTATTTAACGAAGGAGACGTGATTGGCGAAGTTGGAGATAATGATAGTGATGGTTGTAACGGAGTCGGTGGCGAGAGATTTTCGGATTGCGACGGTGATCGGTGAGGAGTTGAGTACGAGGGCGACGAAGGTGGAGTTGTAGTGAGTGGATGAGTAAGAAGTGGGATTGAGATGGGTTTTGTGTTTT

KEG36

GGTTTGTGGATTGTGGGTTTTGTGGTTGTGGGTGTTGTGGTTGTGGGTTGAGTGGAGGAGAAGAATTAGTTGGGTTTGTTTTTGAATTGTGGTTTATAAGAGATTTTTTATTTAACGAAGGAGACGTGATTGGCGAAGTTGGAGATAATGATAGTGATGGTTGTAACGGAGTCGGTGGCGAGAGATTTTCGGATTGCGACGGTGATCGGTGAGGAGTTGAGTACGAGGGCGATGAAGGCGGAGTTGTAGTGAGTGGACGAGTAAGAAGCGGGATTGAGACGGGTTTTGTGTTTT

KEG38

GGTTTGTGGATTGTGGGTTTTGTGGTTGTGGGCGTTGTGGTTGTGGGTTGAGTGGAGGAGAAGAATTAGTTGGGTTTGTTTTTGAATTGTGGTTTATAAGAGATTTTTTATTTAACGAAGGAGACGTGATCGGCGAAGTTGGAGATAATGATAGTGATGGTTGTAACGGAGTCGGTGGCGAGAGATTTTCGGATTGCGACGGTGATCGGTGAGGAGTTGAGTACGAGGGCGACGAAGGTGGAGTTGTAGTGAGTGGACGAGTAAGAAGCGGGATTGAGACGGGTTTTGTGTTTT

KEG39

TGTGGGTGTGAGGAATATGGGTTACAGGTTTTTGGGTTATTTATATGTGGGCGTTGCAGGGGGAGTACGAGTGGAGGAGAAGAATTAGTTGGGTTTGTTTTTGAATCGTGGTTTCTAATAGATTTTTTATTTAACGAAGGAGATGTGATCGGCGAAGTTGGAGATAATGATAGTGATGGTTGTAACGGAGTCGGTGGCGAGAGATTTTCGGATTGCGATGGTGATTGGTGAGGAGTTGAGTACGAGGGTGACGAAGGCGGAGTTGTAGTGAGTGGACGAGTAAGAAGCGGGATTGAGACGGGTTTTGTGTTTT

KEG40

GGTTTGTGGATTGTGGGTTTTGTGGTTGTGGGTGTTGTGGTTGTGGGTTGAGTGGAGGAGAAGAATTAGTTGGGTTTGTTTTTGAATTGTGGTTTATAAGAGATTTTTTATTTAACGAAGGAGACGTGATCGGCGAAGTTGGAGATGATGATAGTGATGGTTGTAACGGAGTCGGTGGCGAGAGATTTTCGGATTGCGACGGTGATCGGTGAGGAGTTGAGTACGAGGGCGACGAAGGCGGAGTTGTAGTGAGTGGACGAGTAAGAAGCGGGATTGAGACGGGTTTTGTGTTTT

KEG42

GGTTTGTGGATTGTGGGTTTTGTGGCTGTGGGCGTTGTGGCTGTGGGCTGAGTGGAGGAGAAGAATTAGTTGGGTTTGTTTTTGAATTGTGGTTTACAAGAGATTTTTCACTCAACGAAGGAGACGTGATCGGCGAAGTTGGAGACAATGACAGTGATGGTTGTAACGGAGTCGGTGGCGAGAGATTTTCGGACTGCGACGGTGACCGGTGAGGAGCTGAGTACGAGGGCGACGAAGGTGGAGTTGTAGTGAGTGGACGAGTAAGAAGCGGGACTGAGACGGGTCTTGTGTTTT

KEG43

GGTTTGTGGATTGTGGGTTTTGTGGTTGTGGGTGTTGTGGTTGTGGGTTGAGTGGAGGAGAAGAATTAGTTGGGTTTGTTTCTGAATTGTGGTTTATAAGAGATTTTTTATTTAACGAAGGAGACGTGATCGGCGAAGTTGGAGATAATGATAGTGATGGTTGTAACGGAGTCGGTGGCGAGAGATTTTCGGATTGTGACGGTGATCGGTGAGGAGTTGAGTACGAGGGCGACGAAGGCGGAGTTGTAGTGAGTGGACGAGTAAGAAGCGGGATTGAGACGGGTTTTGTGTTTT

KEG44

GGTTTGTGGATTGTGGGTTTTGTGGTTGTGGGTGTTGTGGTTGTGGGTTGAGTGGAGGAGAAGAATTAGTTGGGTTTGTTTTTGAATTGTGGTTTATAAGAGATTTTTTATTTAATGAAGGAGATGTGATTGGTGAAGTTGGAGATAATGATAGTGATGGTTGTAATGGAGTTGGTGGCGAGAGATTTTCGGATTGCGACGGTGATCGGTGAGGAGTTGAGTACGAGGGCGACGAAGGTGGAGTTGTAGTGAGTGGATGAGTAAGAAGCGGGATTGAGACGGGTTTTGTGTTTT

***ago4*-EKcr**

AEG10

GGTTTGTGGATTGTGGGTTTTGTGGTTGTGGGTGTTGTGGCTGTGGGCTGAGTGGAGGAGAAGAATCAGTTGGGTCTGTTTTTGAATTGTGGTCTACAAGAGATTTTTTATTCAATGAAGGAGACGTGATCGGCGAAGTTGGAGATAATGACAGTGATGGTTGTAACGGAGTCGGTGGCGAGAGATTTTCGGACTGCGACGGTGACCGGTGAGGAGTTGAGTACGAGGGCGATGAAGGCGGAGTTGTAGTGAGTGGATGAGCAAGGAGCGGGATTGAGACGGGTCTTGTGTTTT

AEG11

GGTTTGTGGATTGTGGGTTTTGTGGTTGTGGGCGTTGTGGTTGTGGGTTGAGTGGAGGAGAAGAATTAGTTGGGTCTGTTTTTGAATTGTGGTTTACAAGAGATTTTTTATTTAACGAAGGAGATGTGATCGGCGAAGTTGGAGATAATGATAGTGATGGTTGTAACGGAGTCGGTGGCGAGAGATTTTCGGACTGCGACGGTGATCGGTGAGGAGTTGAGTACGAGGGTGATGAAGGTGGAGTTGTAGTGAGTGGATGAGTAAGAGGTGGGATTGAGACGGGTTCTGTGTTTT

AEG12

TTTTTCTCCACGCAGAGGTTTGTGGGTGTGGGAAATATGTGTGCGTGGATTGTGTGTTTTGTGTTTGTGGGCGTTGTGTTTGTGGTTTGAGTGGAGGAGAAGAAATAGTGGGGTCTGTTTTTGAATTGTGGTTTACAAGAGATTTTTCACTCAATGAAGGAGACGTGATCGGCGAAGTTGGAGACAATGACAGTGATGGTTGTAACGGAGTCGGTGGCGAGAGATTTTCGGATTGCGACGGTGATCGGTGAGGAGTTGAGTACGAGGGTGATGAAGGTGGAGTTGTAGTGAGTGGACGAGTAAGAAGCGGGATTGAGATGGGTTTTGTGTTTT

AEG15

AAGAAAAAGAACAAGAAGGGTTTTCTTTTGAATACAGTTGTCAACAGGTTTTTATTTATCCAAGGAGGACGAATCGACGGAGGTGGAGATAATGATAATGATGGTTGTTACTGAGTCGGTGGCGAGAGATTTTTTGGTTGCGACGGTCGCCGGTGAGGAGCTGAGCACGAGGGCGACGAAGGCGGAGTTGTAGTGAGTGGACGAGCAAGAAGCGGGACTGAGACGGGTCTTGTGTCTT

AEG17

GGCTTGTGGATTGTGGGTTTTGTGGCTGTGGGCGTTGTGGTTGTGGGTTGAGTGGAGGAGAAGAATCAGTTGGGTTTGTTTTTGAATTGTGGTTTACAAGAGATTTTTTATTCAACGAAGGAGACGTGATCGGCGAAGTTGGAGATAATGACAGTGATGGTTGTAACGGAGTCGGTGGCGAGAGATTTTCGGATTGCGACGGTGATCGGTGAGGAGTTGAGTACGAGGGCGACGAAGGCGGAGTTGTAGTGAGTGGACGAGTAAGAAGCGGGATTGAGACGGGTTTTGTGTTTT

AEG19

GGAGGTGGGTTGAAGGGAGCTCCTTGATCTTCCTTGGTTTTCTACTGGTGTCGGTTTAATGTCTTTGAGTAGAAGAAGAAATTCGTGAGGTTTTTGAATACGGTTTCTTACAGATTATTTTTTTATCTAAGAAGACGACATGAATCGGGAAATTTGGAAATAATGACAGTGATGTTTGTAACGGAGCCGGTGGCGGGAGTTTTTCGGACTGCGACGGTGACCGGTGAGGAGCTGAGTACGAGGGTGACGAAGGTGGAGTTGTAGTGAGTGGATGAGTAAGAAGCGGGACTGAGACGGGTTTTGTGTTTT

AEG8

GTGGAGATATGTTACACTCTTCCTCTGGGCGTCTATATGCATAAATGTAAGTGTTGTCGTGGCCGCCTTCGCAGCGTGAATCAACACCCAGGGCATTAGGGAGACTGGTGTTCTACTAGAGTTTATAAATTTCACGAAGCACACTTGATAGGAGAAGTTGGAGACAATGACAGTGATGGTTATAACGGAGTCGGTGGCGAGAGATTTTCGGACTGCGACGGTGATCGGTGAGGAGCTGAGTACGAGGGCGACGAAGGCGGAGTTGTAGTGAGTGGACGAGCAAGAAGCGGGACTGAGACGGGTTTTGTGTTTT

AEG9

GGTTTGTGGATTGTGGGTTTTGTGGCTGTGGGCGTTGTGGCTGTGGGCTGAGTGGAGGAGAAGAATCAGTTGGGTTTGTTTTTGAATTGTGGTTTACAAGAGATTTTTTATTCAACGAAGGAGACGTGATCGGCGAAGTTGGAGACAATGACAGTGATGGTTGTAACGGAGCCGGTGGCGAGAGATTTTCGGACTGCGACGGTGATCGGTGAGGAGTTGAGTACGAGGGCGACGAAGGCGGAGTTGTAGTGAGTGGACGAGCAAGAAGCGGGATTGAGACGGGTTTTGTGTTTT

***hen1*-EKcr**

HEG63

GGTTTGTGGATTGTGGGTTTTGTGGCTGTGGGCGTTGGGGCTGTGGGCTGAGTGGAGGAGAGGAATCAGTTGTTTTTGTTTCTGATTTGTGTTTTACAAGAGATTTTTTACTTAACGAAGGAGACGTGATCGGCGAAGTTGGAGATAATGATAGTGATGGTTGTAACGGAGTCGGTGGCGAGAGATTTTCGGATTGCGACGGTGATCGGTGAGGAGCTGAGTACGAGGGTGACGATGGCGGAGCTGTAGTGAGTGGACAAGTAAGAAGTGGGATTGAGACGGGTTTTGTGTT

HEG64

GGCTTGTGGATTGTGGGTTTTGTGGCTGTGGGCGTTGTGGCTGTGGGCTGAGTGGAGGAGAAGAATCAGTTGGGTCTGTTTTTGAATTGTGGTTTACAAGAGATTTTTTACTCAGCGAAGGAGACGTGATCGGCGAAGTTGGAGACAATGACAGTGATGGTTGTAACGGAGCCGGTGGCGAGAGATTTTCGGACTGCGACGGTGACCGGTGAGGAGCTGAGTACGAGGGCGACGAAGGCGGAGCTGTAGTGAGTGGACGAGTAAGAAGCGGGATTGAGACGGGTCTTGTGTCT

HEG65

GGTTTGTGGATTGTGGGTTTTGTGGCTGTGGGCGTTGTGGCTGTGGGCTGAGTGGAGGAGGAGAATTAGTTGGGTCTGTTTCTGAATTGTGGTCCACAAGAGATTTTTCATTCGACGAAGGAGACGTGATCGGCGAAGTTGGAGATAATGACAGTGATGGTTGTAACGGAGCCGGTGGCGAGAGATTTTCGGACTGCGATGGTGACCGGTGAGGAGCTGGGTACGAGGGCGACGATGGCGGACCTGTACTGAGTGGACGATCAAGAAGCGGGACCGACACGGGTCTTGTGTTTT

HEG66

GGTTTGTGGATTGTGGGTTTTGTGGCTGTGGGCGTTGTGGCTGTGGGCTGAGTGGAGGAGAAGAATCAGTTGGTTCTGTTTTTGATTTGTGGTTCACAAGAGATTTTTCATTCAACGAAGGAGACGTGATCGGCGAAGTTGGAGATAATGATAGTGATGGTTGTAACTGAGTCGGTGGCGAGAGATTTTCGGATTGCGACGGTGACCGGTGAGGAGCTGAAACTAGGGCGACTAATGCTGAGCTGTATGAGTGGAA

HEG67

GGTTGTGGGTTTGAGGATGTGGGTTTGTGGATTGTGGGTTTTGTGGCTGTGGGCGTTGTGGATGTAGAATAAGAGGAGGGGTGGTAGGGA

HEG68

GGTTGTGGGTTTAGGAAGTGGGTTTGTGGATTTGTGAGAATAAGATAAAAGAAAAATGGAAAAGAGAAGAAAAAAAAGTGGTGGTATGGAATCTGTTACAGCTATC

HEG69

GGTTTGTGGATTGTGGGTTTTGTGGCTGTGGGCGTTGTGGCTGTGGGCTGAGTGGAGGAGAAGAATCAGTTGGGTTTGTTTTTTTATTGTGGTTTGCAAGAGATTTTTCACTCAACGAAGGAGACGTGATCGGCGAAGTTGGAGACAGTGACAGTGATGGTTGTAACGGAGTCGGTGGCGAGAGATTTTCGGATTGCGACGGTGATCGGTGGGGAGCTGAGTACGGGGGCGACGAAGGCGGAGCTGTAGTGAGTGGACGAGCAAGAAGCGGGACTGAGACGGGTCTTGTGTTTT

HEG71

GGCTTGTGGATTGTGGGTTTTGTGGTTGTGGGCGTTGTGGCTGTGGGCTGAGTGGAGGAGAAGAATCGGTTGGGTCTGTTTTTGAATTGTGGTTTATAAGAGATTTTTTATTTAACGAAGGATACGTGATCGGCGAAGTTGGAGATAATGACATTGATGGTTGTTTCGGAGTCGGTGGCGAGAGACTTTCGGATTGTGACGGTGACCGGTGAGGAGCTGAGTACTAGGGCTACTATTGCTGAGCTGTAGTGAGTGTACGAGTATGAATCGGGACTGAGATGGGTTTTGTGTTTTTTG

HEG73

GGTTTGTGGATTGTGGGTTTTGTGGCTGTGGGCGTTGTGGCTGTGGGCTGAGTGGAGGAGAAGAATTAGTTGGGTTTGTTTCTGAATTGTGGTTTATAAGAGATTTTTTATTTAACGAAGGAGACGTGATCGGCGAAGTTGGAGATAATGACAGTGATGGTTGTAACGGAGTCGGTGGCGAGAGATTTTCGGACTGCGACGGTGATCGGTGAGGAGTTGAGTACGAGGGCGACGAAGGCGGAGTTGTAGTGAGTGGACGAGTAAGAAGCGGGATTGAGACGGGTTTTGTGTTT

HEG74

GTGGGGAATGTTGTGGATTTGGGTTATTTGGTTGTGGGTTTGAGGAGTGTGGGTTTGTGGATTGGGGGTTTGGTGGCTGGAGACGATGTGGCTGGGTCTGTTTTTGAATTGTGGTTTACAAGAGATTTTTTATTTAACGAAGGAGACGTGATCGGCGAAGTTGGAGACAATGACAGTGATGGTTGTAACGGAGTCGGTGGCGAGAGATTTTCGGATTGCGACGGTGATCGGTGAGGAGTTGAGTACGAGGGCGACGAAGGCGGAGTTGTAGTGAGTGGACGAGTAAGAAGCGGGATTGAGACGGGTTTTGTGTT

***rdr2*-EKcr**

REG90

GGTTTGTGGATTGTGGGTTTTGTGGCTGTGGGCGTTGTGGCTGTGGGCTGAGTGGAGGAGAAGAATTAGTTGGGTTTGTTTTTGAATTGTGGTTTATAAGAGATTTTTTATTCAACGAAGGAGATGTGATTGGTGAAGTTGGAGATAATGACAGTGATGGTTGTAACGGAGTCGGTGGCGAGAGATTTTTGGACTGCGACGGTGACCGGTGAGGAGCTGAGTACGAGGGCGACGAAGGCGGAGCTGTAGTGAGTGGACGAGTAAGAAGCGGGATTGAGACGGGTTTTGTGTTTT

REG91

GGTCTGTGGATTGTGGGTTTTGTGGCTGCGGGCGTTGTGGCTGTGGGCTGAGTGGAGGAGAAGAATCAGTTGGGTTTGTTTTTGAATTGTGGTTTACAAGAGATTCTTCACTCAACGAAGGAGACGTGATCAGCGAAGTTGGAGATAATGACAGTGATGGTTGTAACGGAGTTGGTGGCGAGAGATTTTCGGACTGCGACGGTGATCGGTGAGGAGCTGAGTATGAGGGCGACGAAGGTGGAGCTGTAGTGAGTGGACGAGCAAGAAGCGGGATTGAGACGGGTTTTGTGTTTT

REG92

GGTTTGTGGATTGTGGGTTTTGTGGCTGTGGGCGTTGTGGTTGTGGGTTGAGTGGAGGAGAAGAATCAGTTGGGTCTGTTTCTGAATTGTGGTCCACAAGAGATTTTTCACTCAACGAAGGAGACGTGATCGGCGAAGTTGGAGATAATGACAGTGATGGTTGTAACGGAGTCGGTGGCGAGAGATTTTCGGATTGCGACGGTGATCGGTGAGGAGCTGAGTACGAGGGCGACGAAGGTGGAGTTGTAGTGAGTGGACGAGCAAGAAGCGGGATTGAGACGGGTTTTGTGTTTT

REG93

GGTTTGTGGATTGTGGGTTTTGTGGTTGTGGGTGTTGTGGTTGTGAGTTGAGTGGAGGAGAAGAATTAGTTGGGTCTGCTTTTGAATTGTGGTTTATAAGAGATTTTTCACTCAACGAAGGAGACGTGATCGGCGAAGTTGGAGACAATGACAGTGATGGTTGTAATGGAGTCGGTGGCGAGAGATTTTCGGACTGCGACGGTGACCGGTGAGGAGTTGAGTACGAGGGCGACGAAGGCGGAGCTGTAGTGAGTGGACGAGCAAGAAGCGGGATTGAGACGGGTCTTGTGTCTT

REG95

GGTTTGTGGATTGTGGGTTTTGTGGCTGTGGGTGTTGTGGTTGTGGGTTGAGTGGAGGAGAAGAATTAGTTGGGTTTGTTTTTGAATTGTGGTTTACAAGAGATTTTTTATTCAACGAAGGAGACGTGATCGGCGAAGTTGGAGACAATGACAGTGATGGTTGTAACGGAGTCGGTGGCGAGAGATTTTCGGACTGCGACGGTGACCGGTGAGGAGCTGAGTACGAGGGCGACGAAGGCGGAGTTGTAGTGAGTGGACGAGCAAGAAGCGGGATTGAGACGGGTTTTGTGTTTT

REG96

GGTTTGTGGATTGTGGGTTTTGTGGCTGTGGGCGTTGTGGCTGTGGGCTGAGTGGAGGAGAAGAATCAGTTGGGTTTGTTTTTGGATTGTGGTTTACAAGAGATTTTTCACTCAACGAAGGAGACGTGATCGGCGAAGTTGGAGATAATGACAGTGATGGTTGTAACGGAGCCGGTGGCGAGAGATTTTCGGACTGCGACGGTGACCGGTGAGGAGCTGAGTACGAGGGCGACGAAGGCGGAGCTGTAGTGAGTGGACGAGCAAGAAGCGGGACTGAGACGGGTCTTGTGTTTT

REG97

GGTTTGTGGATTGTGGGTTTTGTGGTTGTGGGTGTTGTGGTTGTGGGTTGAGTGGAGGAGAAGAATTAGTTGAGTTTGTTTTTGAATTGTGGTTTATAAGAGATTTTTTATTTAACGAAGGAGACGTGATCGGTGAAGTTGGAGATAATGATAGTGATGGTTGTAACGGAGTCGGTGGCGAGAGATTTTCGGACTGCGACGGTGATCGGTGAGGAGTTGAGCACGAGGGCGACGAAGGCGGAGTTGTAGTGAGTGGACGAGTAAGAAGCGGGATTGAGATGGGTTTTGTGTTTT
